# Functional characterization of a xylose transporter in *Aspergillus nidulans*

**DOI:** 10.1186/1754-6834-7-46

**Published:** 2014-04-01

**Authors:** Ana Cristina Colabardini, Laure Nicolas Annick Ries, Neil Andrew Brown, Thaila Fernanda dos Reis, Marcela Savoldi, Maria Helena S Goldman, João Filipe Menino, Fernando Rodrigues, Gustavo Henrique Goldman

**Affiliations:** 1 Departamento de Ciências Farmacêuticas, Faculdade de Ciências Farmacêuticas de Ribeirão Preto, Universidade de São Paulo, Av. do Café S/N, CEP 14040-903 Ribeirão Preto, São Paulo, Brazil; 2 Faculdade de Filosofia, Ciências e Letras de Ribeirão Preto, Universidade de São Paulo, São Paulo, Brazil; 3 ICVS/3B’s - PT Government Associate Laboratory, Braga/Guimarães, Portugal and Life and Health Sciences Research Institute (ICVS), School of Health Sciences, University of Minho, Braga, Portugal; 4 Laboratório Nacional de Ciência e Tecnologia do Bioetanol – CTBE, Caixa Postal 6170 13083-970, Campinas, São Paulo, Brazil

**Keywords:** *Aspergillus nidulans*, xylose transporters, *Saccharomyces cerevisiae*, second generation bioethanol

## Abstract

**Background:**

The production of bioethanol from lignocellulosic feedstocks will only become economically feasible when the majority of cellulosic and hemicellulosic biopolymers can be efficiently converted into bioethanol. The main component of cellulose is glucose, whereas hemicelluloses mainly consist of pentose sugars such as D-xylose and L-arabinose. The genomes of filamentous fungi such as *A. nidulans* encode a multiplicity of sugar transporters with broad affinities for hexose and pentose sugars. *Saccharomyces cerevisiae*, which has a long history of use in industrial fermentation processes, is not able to efficiently transport or metabolize pentose sugars (e.g. xylose). Subsequently, the aim of this study was to identify xylose-transporters from *A. nidulans*, as potential candidates for introduction into *S. cerevisiae* in order to improve xylose utilization.

**Results:**

In this study, we identified the *A. nidulans xtrD* (*x*ylose *tr*ansporter) gene, which encodes a Major Facilitator Superfamily (MFS) transporter, and which was specifically induced at the transcriptional level by xylose in a XlnR-dependent manner, while being partially repressed by glucose in a CreA-dependent manner. We evaluated the ability of *xtrD* to functionally complement the *S. cerevisiae* EBY.VW4000 strain which is unable to grow on glucose, fructose, mannose or galactose as single carbon source. In *S. cerevisiae*, XtrD was targeted to the plasma membrane and its expression was able to restore growth on xylose, glucose, galactose, and mannose as single carbon sources, indicating that this transporter accepts multiple sugars as a substrate. XtrD has a high affinity for xylose, and may be a high affinity xylose transporter. We were able to select a *S. cerevisiae* mutant strain that had increased xylose transport when expressing the *xtrD* gene.

**Conclusions:**

This study characterized the regulation and substrate specificity of an *A. nidulans* transporter that represents a good candidate for further directed mutagenesis. Investigation into the area of sugar transport in fungi presents a crucial step for improving the *S. cerevisiae* xylose metabolism. Moreover, we have demonstrated that the introduction of adaptive mutations beyond the introduced xylose utilization genes is able to improve *S. cerevisiae* xylose metabolism.

## Background

Efforts to mitigate global warming and reduce fossil fuel consumption, while sustaining future world-wide energy demands has substantially increased investment in the development of alternative energy sources such as bioethanol. Currently, bioethanol production throughout the world is based on first-generation technologies (1G) that ferment simple sugars derived from high-sugar-containing plants such as sucrose from sugarbeet or sugarcane and starch from corn. During these processes the remaining carbon locked within the lignocellulosic substrate is not converted into bioethanol. In the case of sugarcane, the waste material, termed bagasse, contains a third of the energy stored within the plant. If bagasse could also be used to produce bioethanol instead of being burnt, production could increase by 40% [1,2]. Thus, the utilization of non-food lignocellulosic plant residues for bioethanol production by second-generation technologies (2G) is extremely attractive for the biofuel industry, representing both economic and environmental gains via reducing the carbon footprint and production costs [3,4].

Lignocellulosic biomass generally consists of cellulose (40% to 50%), hemicelluloses (25% to 35%) and lignin (15% to 20%) [5]. The objective of 2G technologies is to dually utilize the hexose and pentose sugars extracted from cellulose and hemicelluloses in the production of bioethanol, therefore making the process economically viable [6,7]. The hexose sugar glucose represents 60% of the total sugars found in cellulose, whereas hemicellulose is predominantly composed of pentose sugars such as D-xylose and L-arabinose [7]. The conversion of lignocellulose into ethanol, therefore, requires an organism capable of fermenting both hexose and pentose sugars [8]. *Saccharomyces cerevisiae* is the preferred microbe utilized for industrial fermentation processes including bioethanol production [9,10]. Although the *S. cerevisiae* genome appears to encode all components necessary for xylose metabolism, these components are not efficient to allow growth on xylose as the sole carbon source [10].

After xylose is taken up into the cell by the microorganism, it is first converted to xylitol, which is then converted to phosphorylated xylulose prior to generating the pentose phosphate pathway intermediate xylulose-5-phosphate. Xylose reductase (XR) and xylitol dehydrogenase (XDH) catalyze the first two reactions in this pathway and their activity strictly depends on the respective cofactors nicotinamide adenine dinucleotide phosphate (NADPH) and nicotinamide adenine dinucleotide (NAD)^+^. During aerobic respiration, an excess of reduced nicotinamide adenine dinucleotide (NADH) is re-oxidized, but during anaerobic respiration, NADH accumulates in the cell and xylose utilization is slowed down. The XR of the natural xylose-assimilating yeast *Scheffersomyces stipitis* uses NADH almost as well as NADPH, avoiding an imbalance of cofactors [9]. Significant efforts have been made to engineer *S. cerevisiae* strains with improved xylose metabolism. Recombinant *S. cerevisiae* strains are able to metabolize xylose and ferment xylulose through the heterologous expression of XR-XDH or other enzymes such as xylose isomerase, a bacterial enzyme that catalyzes the one-way conversion of xylose to xylulose [11-15].

However, another limiting step in the utilization of xylose by *S. cerevisiae* is the transport of xylose into the cell. Domestic and wild-type *S. cerevisiae* species transport xylose into the cell with low-affinity (K_M_ = 100 mM to 190 mM) via the expression of native high-affinity hexose transporters, such as *GAL2* and *HXT7*[7,16]. A functional survey of the expression of heterologous sugar transporters in recombinant *S. cerevisiae* evaluated 26 monosaccharide transporters*.* Ten of these transporters conferred growth on individual sugars, while the majority exhibited a broad substrate range, with all of them favoring glucose transport when in competition with other monosaccharides. Glucose has previously been shown to inhibit xylose transport [7,10]. Therefore, heterologous expression of a specific xylose transporter, especially those transporting xylose with higher affinity than glucose [7,10], is indispensable for improved xylose utilization in recombinant *S. cerevisiae*. Alternatively, transporter proteins can be engineered through introducing mutations, in order to have increased xylose transport in *S. cerevisiae*[7,10].

In contrast to *S. cerevisiae*, filamentous fungi are specialized in lignocellulosic biomass degradation and through the secretion of a large repertoire of hydrolytic enzymes, the sugar polymers are broken down into simple sugars which are subsequently taken up into the cell [17,18]. The genomes of filamentous fungi also encode large numbers of sugar transporters. However, very few fungal sugar transporters have been functionally characterized. *Aspergilli* are a group of filamentous fungi capable of producing a wide variety of plant biomass-degrading enzymes and can grow on lignocellulose. In *Aspergilli* the lignocellulose utilization pathway is tightly repressed by the transcription factor CreA that mediates carbon catabolite repression (CCR) and positively induced by the regulon-specific transcription factors XlnR, ClrA, and ClrB [17,18]. The aim of this study was therefore to identify xylose transporter-encoding genes in *Aspergillus nidulans* that are potential candidates for heterologous gene expression in *S. cerevisiae*. We identified the *A. nidulans xtrD* (*x*ylose *tr*ansporter) gene, encoding a transporter from the major facilitator superfamily (MFS). Induction of *xtrD* at the transcriptional level was observed in the presence of xylose in an XlnR-dependent manner. We also showed that in *A. nidulans xtrD* is repressed by glucose in a CreA-dependent manner. To further characterize the protein encoded by *xtrD*, we expressed the gene in a *S. cerevisiae* strain that is unable to grow on glucose, fructose, mannose or galactose as a single carbon source. In *S. cerevisiae* XtrD was targeted to the plasma membrane and its expression was able to restore growth on xylose, glucose, galactose, and mannose as single carbon sources, indicating that this transporter accepts multiple sugars as a substrate. This work identified an efficient xylose transporter which could potentially be used in future studies to improve *S. cerevisiae* xylose uptake through site-directed mutagenesis, directed evolution or in combination with other heterologous transporters.

## Results

### Xylose metabolism is partially repressed by glucose

**Figure 1:**
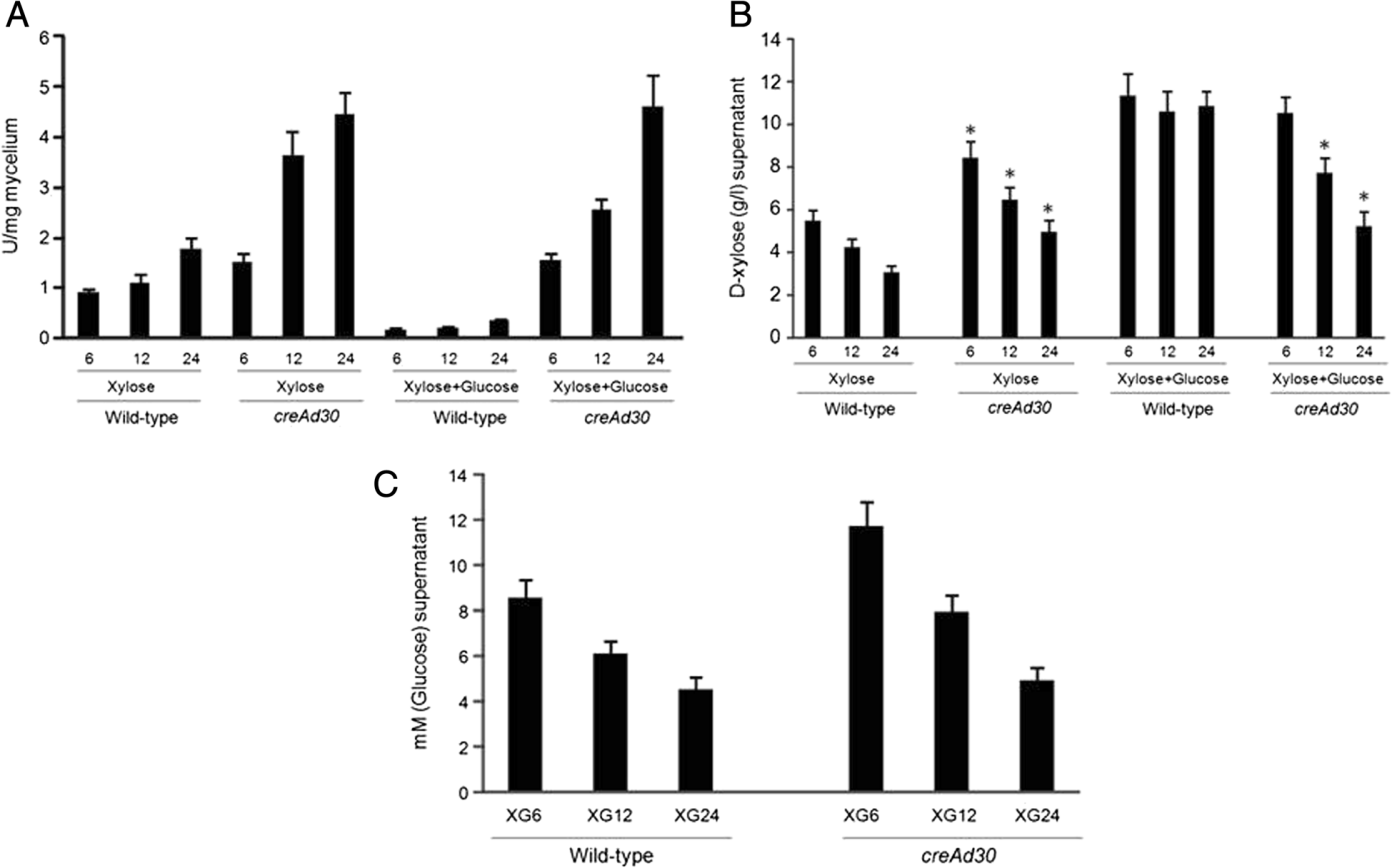
***A. nidulans *growth in the presence of xylose or xylose plus glucose. (A)** Enzymatic activity of endo-1,4-β-xylanase in the culture supernatant in the presence of xylose (X) or xylose plus glucose (XG) after 6 h, 12 h and 24 h incubation at 37°C. One unit of enzyme activity is defined as the amount of enzyme required to release 1 μmol of D-xylose reducing-sugar equivalents from arabinoxylan, at pH 4.5 per minute at 40°C. Error bars represent the standard deviation for three biological replicates; *significant difference in the *P*-value (<0.01, one-way analysis of variance (anova) and Newman-Keuls test) between X and XG-grown cultures for each strain. **(B)** Xylose concentrations in the supernatant of *A. nidulans* wild-type and *creAd30* cultures grown in the presence of xylose (X) or xylose plus glucose (XG) for 6 h, 12 h, and 24 h at 37°C. **(C)** Glucose concentrations in the supernatant of *A. nidulans* wild-type and *creAd30* cultures grown in the presence of xylose or xylose plus glucose for 6 h, 12 h and 24 h at 37°C. Error bars represent the standard deviation for three biological replicates; *significant difference in the *P*-value (<0.01, one-way Anova and Newman-Keuls test) between X and XG-grown cultures for each strain.

Initially to establish the parameters for the induction of xylose transporters and xylose uptake, the *A. nidulans* wild-type and carbon catabolite resistant *creAd30* strains were grown on xylose as a sole carbon source or in media containing both xylose and glucose. The *A. nidulans* cultures were first grown on fructose and then transferred to media containing 1% xylose or media containing 1% xylose plus 1% glucose for 6, 12 and 24 h (Figure 1). The endoxylanase activity of the supernatant was recorded for both strains during all time points (Figure 1A). In the wild-type strain, xylose clearly induced the secretion of endoxylanases, whereas the simultaneous presence of xylose and glucose reduced endoxylanase production (Figure 1A). On the other hand, in the *creAd30* strain no such difference was observed between the xylose and the xylose plus glucose cultures for most time points (Figure 1A). Also, the *creAd30* mutant secreted more endoxylanases than the wild-type strain (Figure 1A). In order to know whether this observation depends on the amount of internalized xylose and glucose in both strains, the concentration of the respective sugars in the extracellular media was determined (Figure 1B and 1C). The rate of xylose uptake was faster in the wild-type strain than in the *creAd30* mutant when grown on xylose as a sole carbon source (Figure 1B). During growth of the wild-type strain in the simultaneous presence of xylose and glucose, a high concentration of xylose persisted in the extracellular medium (Figure 1B). In contrast, in the carbon catabolite derepressed *creAd30*-strain xylose was taken up even in the presence of glucose (Figure 1B), whereas glucose uptake was slightly slower in the *creAd30* strain (Figure 1C). Therefore, *A. nidulans* preferentially takes up glucose, with xylose transport and metabolism being partially subjected to CCR.

### Transcriptional profiling of *A. nidulans* in the presence of xylose

**Figure 2:**
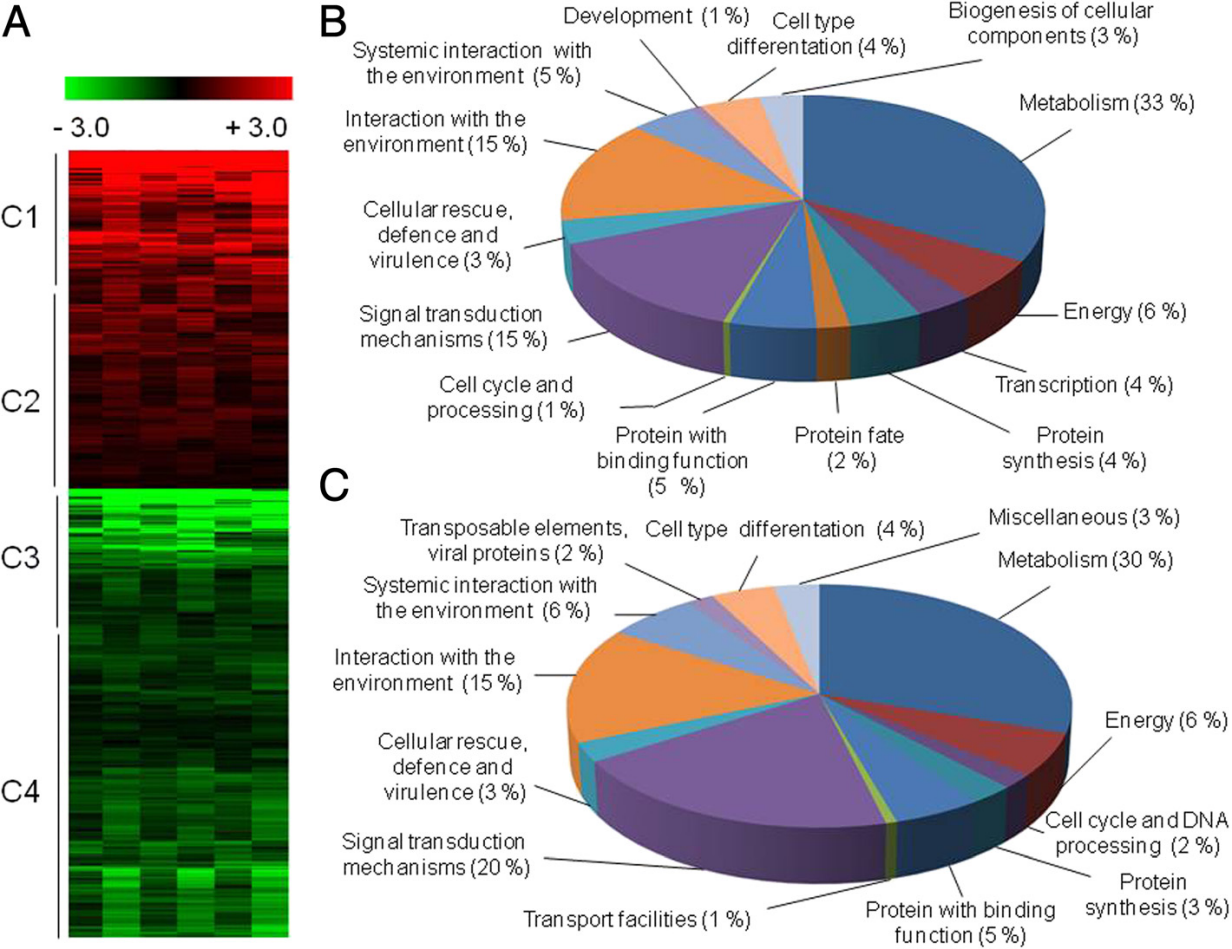
**Gene expression patterns of *A. nidulans *wild-type cultures grown in the presence of xylose. (A)** Microarray results: the color code (red = expression, green = repression) displays the log_2_ (Cy5/Cy3) value for each time point with Cy3 as the reference value (time point = 0, growth on fructose). The data were visualized based on similar expression vectors using Euclidean distance and hierarchical clustering with average linkage clustering and classed into four clusters (C1 to C4). **(B**, **C)** Classification of genes from **(A)** into the respective FunCat categories [19].

Genome-wide transcriptional profiling was utilized to identify the genes and pathways involved in xylose transport and metabolism. The *A. nidulans* wild-type strain was grown in media containing 1% fructose (reference) and then transferred to media containing 1% xylose for 6, 12 and 24 h. The main objective was to identify genes with increased expression in xylose when compared to the control condition. In total 1,558 genes were differentially expressed (*P* < 0.01) in at least one time point (GSE50485; see Additional file 1). The proteins encoded by differentially expressed genes were classified into FunCat functional categories (http://mips.helmholtz-muenchen.de/proj/funcatDB/search_main_frame.html), revealing a wide variety of cellular processes. Hierarchical clustering allowed the classification of these genes into four main clusters (Figure 2A), where genes from clusters C1 and C2 were induced, whilst genes from clusters C3 and C4 were repressed, during growth on xylose. All four clusters were enriched (*P* < 0.001) in genes encoding proteins with functions in metabolism, signal transduction mechanisms, interaction with the environment, and energy (Figures 2B and 2C).

As expected, there was high upregulation of genes involved in xylose metabolism, such as D-xylose reductase (AN9064), xylitol dehydrogenase (AN9064) and xylulokinase (AN8790). In addition, there was an induction of xylanases and other plant cell-wall-degrading enzyme-encoding genes (Tables 1 and 2). The differentially expressed genes that encode putative transporters and transcription factors are shown in Table 3. One of these genes is *abaA*, which encodes a transcriptional activator involved in the regulation of conidiation and which is required for phialide differentiation [20] (Table 3). We did not observe any conidiation in our cultures (data not shown) and it remains to be investigated whether *abaA* plays a role in gene regulation during growth of *A. nidulans* in the presence of xylose. Multiple transporter-encoding genes resided in cluster C1 and were highly upregulated when *A. nidulans* mycelia were transferred to xylose containing media (Table 3). Among the several putative transporters, we randomly selected five transporter-encoding genes for further study (AN6412 (*xtrA*), AN3264 (*xtrB*), AN2358 (*xtrC*), AN0250 (*xtrD*) and AN4148 (*xtrE*)). The induction of the five transporter encoding genes was confirmed by RT-qPCR. All five genes were induced to a different extent in the presence of xylose and repressed in the presence of glucose (Table 4). Therefore, xylose-specific expression profiling has identified several *A. nidulans* transporter-encoding genes, which may represent potential xylose transporters.

**Table 1:** **Differentially expressed genes, predicted to encode proteins involved in carbon metabolism, between 24 h fructose and 6 h, 12 h and 24 h xylose in *A. nidulans***

**Function**	**ID**	**Description**			
			**6 h**	**12 h**	**24 h**
			**log**_**2 **_**Cy5/Cy3**	**log**_**2 **_**Cy5/Cy3**	**log**_**2 **_**Cy5/Cy3**
**C1 category**					
*xyrA*	AN0423	Putative D-xylose reductase	7.75	7.36	7.17
*acuD*	AN5634	Isocitrate lyase	1.90	1.68	2.24
*gfdB*	AN6792	Putative NAD + dependent glycerol 3-phosphate dehydrogenase	1.30	1.93	1.32
*maeA*	AN6168	Putative malate dehydrogenase	2.05	2.12	2.19
AN0443	AN0443	Alcohol dehydrogenase	1.16	0.65	0.99
AN1274	AN1274	Alditol:NADP^+^ oxidoreductase; role in arabinose and xylose catabolic process	3.98	3.11	2.81
AN2099	AN2099	alternative oxidase AoxA	2.03	1.36	2.21
AN2951	AN2951	Putative UDP-glucose 4-epimerase; role in galactose and galactitol metabolism	1.07	1.06	2.01
AN3432	AN3432	Putative epimerase with a predicted role in carbohydrate metabolism	3.84	3.43	3.57
AN7343	AN7343	Amylase cluster transcriptional regulator AmyR	1.21	0.80	1.41
AN8790	AN8790	Putative xylulokinase	2.28	1.82	1.51
AN9031	AN9031	Fumarylacetoacetate hydrolase	1.25	1.04	1.34
AN9064	AN9064	Xylitol dehydrogenase	3.49	2.68	2.37
AN9152	AN9152	NAD dependent epimerase/dehydratase family protein	1.50	0.96	2.14
AN9457	AN9457	L-galactose dehydrogenase	6.05	4.58	4.86
**C2 category**					
ladC	AN8552	L-arabinitol 4-dehydrogenase (PPP)	0.32	0.15	0.17
orlA	AN3441	Trehalose 6-phosphate phosphatase	0.68	0.39	0.37
ugeA	AN4727	UDP-glucose 4-epimerase	0.78	1.07	0.54
AN10783	AN10783	6-phosphogluconate dehydrogenase family protein	0.99	1.15	0.62
AN2470	AN2470	Alcohol dehydrogenase	0.79	0.66	1.13
AN7588	AN7588	Putative ribulose-phosphate 3-epimerase (PPP)	0.79	0.39	0.58
AN8707	AN8707	Putative fumarate dehydratase with a predicted role in the TCA cycle	0.60	0.46	0.13
AN9347	AN9347	Alcohol dehydrogenase	0.76	0.60	1.02
**C3 category**					
acoA	AN5525	Putative aconitate hydratase with a predicted role in the TCA cycle	-0.62	-0.66	-0.55
alcS	AN8981	Protein with homology to GPR1/FUN34/YaaH family members	-2.80	-2.73	-2.85
carC	AN8793	Putative succinate dehydrogenase (ubiquinone)	-0.40	-0.31	-0.52
galF	AN9148	Putative UTP-glucose-1-phosphate uridylyltransferase	-0.48	-0.20	-0.54
gpgA	AN2742	Gamma subunit of a heterotrimeric G protein composed of FadA	-0.34	-0.36	-0.42
gprC	AN3765	Putative G-protein coupled receptor	-1.59	-2.83	-3.14
gsdA	AN2981	Putative glucose 6-phosphate 1-dehydrogenase (PPP)	-0.49	-0.55	-0.88
idpA	AN2999	Isocitrate dehydrogenase (NADP+) with a predicted role in the TCA cycle	-0.96	-1.10	-0.91
mcsA	AN6650	Methylcitrate synthase with a predicted role in the TCA cycle	-1.31	-1.05	-2.05
mdhA	AN6717	Putative malate dehydrogenase with a predicted role in the TCA cycle	-0.48	-0.41	-0.51
pycA	AN4462	Putative pyruvate carboxylase or glutathione synthase	-0.34	-0.20	-0.66
sfaD	AN0081	Beta subunit of a heterotrimeric G protein composed of FadA	-0.40	-0.52	-0.61
tpiA	AN6900	Putative triose-phosphate isomerase with a role in gluconeogenesis and glycolysis	-0.71	-0.74	-0.66
vmaB	AN6232	Putative F1F0-ATPase complex subunit with a predicted role in energy metabolism	-0.83	-0.86	-0.90
AN0252	AN0252	Putative F1F0-ATPase complex subunit with a predicted role in energy metabolism	-0.69	-0.54	-0.70
AN0567	AN0567	Putative alcohol oxidase with a predicted role in glycerol metabolism	-2.13	-1.77	-2.73
AN0896	AN0896	Putative succinate dehydrogenase	-0.78	-0.64	-0.66
AN1534	AN1534	Putative F1F0-ATPase complex subunit with a predicted role in energy metabolism	-0.53	-0.52	-0.44
AN2208	AN2208	Putative galactose 1-dehydrogenase	-0.65	-0.85	-0.97
AN2315	AN2315	Putative F1F0-ATPase complex subunit with a predicted role in energy metabolism	-0.47	-0.43	-0.57
AN2316	AN2316	Putative cytochrome c oxidase subunit with a predicted role in energy metabolism	-0.61	-0.52	-0.52
AN2815	AN2815	Putative mannitol 2-dehydrogenase with a predicted role in mannose/mannitol	-0.62	-0.91	-0.45
AN3088	AN3088	Putative F1F0-ATPase complex subunit with a predicted role in energy metabolism	-0.39	-0.40	-0.27
AN4525	AN4525	Putative cytochrome c oxidase subunit with a predicted role in energy metabolism	-0.68	-0.74	-0.55
AN5629	AN5629	Putative NADH dehydrogenase (ubiquinone) with a predicted role in energy metabolism	-0.45	-0.52	-0.73
AN5703	AN5703	Electron-transferring-flavoprotein dehydrogenase with a predicted role in energy metabolism	-0.44	-0.50	-0.56
AN5907	AN5907	Putative ribose-5-phosphate isomerase	-0.96	-1.00	-1.13
AN6077	AN6077	Putative NADH dehydrogenase (ubiquinone)	-0.52	-0.56	-0.53
AN6287	AN6287	F1F0-ATPase complex subunit with a predicted role in energy metabolism	-0.59	-0.59	-0.59
AN8118	AN8118	Putative cytochrome c oxidase subunit with a predicted role in energy metabolism	-0.54	-0.57	-0.36
AN8273	AN8273	Putative ubiquinol-cytochrome-c reductase	-0.69	-0.87	-0.73
AN8819	AN8819	Putative dehydrogenase with a predicted role in carbohydrate metabolism	-0.90	-1.03	-1.09

**Table 2:** **Differentially expressed genes, encoding plant cell-wall-degrading enzymes, between 24 h fructose and 6 h, 12 h and 24 h xylose in *A. nidulans***

**Function**	**ID**	**Description**			
			**6 h**	**12 h**	**24 h**
			**log**_**2 **_**Cy5/Cy3**	**log**_**2 **_**Cy5/Cy3**	**log**_**2 **_**Cy5/Cy3**
**C1 category**					
afcC	AN10376	Putative alpha-fucosidase	2.08	1.26	1.60
aguA	AN9286	Protein with alpha-glucuronidase activity	3.48	3.85	2.34
AN0280	AN0280	Putative alpha-1,4-glucosidase	4.34	4.86	4.79
AN0551	AN0551	Putative mannosyl-oligosaccharide 1,2-alpha-mannosidase	0.72	0.77	1.45
AN10375	AN10375	Beta-glucosidase	4.63	3.91	2.35
AN2533	AN2533	Putative alpha-L-arabinofuranosidase	1.10	2.19	1.26
AN8369	AN8369	Glycosyl transferase, group 1 family protein	1.27	0.75	1.39
AN8477	AN8477	Putative beta-1,4-xylosidase	4.41	4.42	2.75
axeA	AN6093	Protein with acetylxylan esterase activity	3.11	3.19	2.86
axhA	AN7908	Protein with alpha-arabinofuranosidase activity	5.26	4.94	5.70
bxlC	AN1477	Putative beta-1,4-xylosidase	5.62	5.43	5.07
bxlC	AN2217	Putative beta-1,4-xylosidase	1.44	1.21	0.39
bxlD	AN7864	Putative beta-1,4-xylosidase	5.98	6.00	6.11
dfgB	AN8421	Putative endo-mannanase GH76 family protein	1.43	2.05	2.21
xlnA	AN3613	Protein with endo-1,4-beta-xylanase activity	5.50	5.70	6.48
xlnB	AN9365	Protein with endo-1,4-beta-xylanase activity	4.03	3.99	2.85
xlnC	AN1818	Endo-1,4-beta-xylanase activity	6.87	7.01	6.54
xlnD	AN2359	Protein with beta-xylosidase	5.22	5.13	5.52
**C2 category**					
AN3740	AN3740	Amidohydrolase family protein	0.91	0.85	0.95
AN5748	AN5748	Putative mannosyl-oligosaccharide	0.36	0.32	0.35
AN10124	AN10124	Beta-glucosidase, putative	0.63	0.52	0.74
agdD	AN7505	Protein with alpha-xylosidase activity	1.00	0.59	0.81
bglQ	AN10127	Putative beta-glucosidase	0.62	0.83	0.36
pelB	AN2569	Protein with pectin lyase activity	0.88	0.81	0.92
pmeA	AN3390	Protein with pectinesterase activity	0.40	0.70	0.58
**C3 category**					
agdB	AN8953	Alpha-glucosidase with a predicted role in maltose metabolism	-3.62	-3.34	-4.09
exgA	AN1332	Putative glycosyl hydrolase, GH5	-0.50	-1.27	-1.79
gelB	AN0558	Putative glycosyl hydrolase, GH72	-0.81	-1.01	-1.04

**Table 3:** **Differentially expressed genes, encoding putative transporters and transcription factors, between 24 h fructose and 6 h, 12 h and 24 h xylose in *A. nidulans***

**Function/ID**	**Description**			
		**6 h**	**12 h**	**24 h**
		**log**_**2 **_**Cy5/Cy3**	**log**_**2 **_**Cy5/Cy3**	**log**_**2 **_**Cy5/Cy3**
**Transporters (C1 category)**				
AN0250	Major facilitator superfamily (MFS) sugar transporter	5.75	5.05	6.05
AN0332	MFS transporter	2.90	1.40	1.25
AN0601	MFS transporter	1.63	1.01	1.84
AN2358	MFS protein	1.84	1.75	2.11
AN2746	MFS monocarboxylate transporter	1.31	0.48	1.31
AN2959	MFS transporter	0.75	1.45	1.70
AN3264	MFS transporter	2.84	1.63	4.13
AN4148	MFS monosaccharide transporter	2.94	3.32	3.39
AN4374	MFS transporter	0.72	1.28	1.36
AN6412	MFS transporter	1.90	1.66	2.90
AN6779	ABC transporter	1.44	1.29	0.68
AN8347	Hexose transporter protein	5.97	5.81	6.08
AN9165	MFS transporter	1.70	2.22	2.32
AN9173	MFS glucose transporter	2.38	1.52	1.66
**Transcription factors (C1 category)**				
AN0273	C2H2 type zinc finger domain-containing protein	0.77	0.93	1.37
AN0422	abaA; TEA/ATTS domain transcriptional activator	4.48	4.22	4.15
AN11169	C6 transcription factor	0.86	0.90	1.27
AN1729	prnA; Transcriptional activator from the zinc binuclear cluster family	1.08	0.68	1.29
AN7170	HLH DNA binding domain protein	1.00	0.59	0.98
AN7343	amylase cluster transcriptional regulator AmyR	1.21	0.80	1.41
**Transporters (C3 category)**				
AN0010	Putative amino acid transporter	-1.74	-3.76	-1.67
AN1797	*S. cerevisiae* ortholog RGT2 has role in glucose transport	-0.42	-1.35	-1.22
**Transcription factors (C3 category)**				
AN2016	amyR; Zn (2)Cys (6) TF involved in starch metabolism	-1.55	-1.68	-1.89

**Table 4:** **Expression values (as determined by qRT-PCR) for the five transporter-encoding *xtrA-E *** **genes when *A. nidulans *** **was grown in fructose for 24 h and then transferred to xylose (X) or xylose and glucose (X + G)-rich media for 6 h, 12 h and 24 h**

	**Control**	**X6**	**X + G6**	**X12**	**X + G12**	**X24**	**X + G24**
***xtrA *** **(AN6412)**	0.55 ± 0.04	0.42 ± 0.06	0.70 ± 0.16	4.14 ± 0.21	0.66 ± 0.09	7.03 ± 0.35	1.71 ± 0.02
***xtrB *** **(AN3264)**	0.003 ± 0.002	0.003 ± 0.00	0.010 ± 0.002	0.033 ± 0.003	0.004 ± 0.00	0.013 ± 0.002	0.003 ± 0.00
***xtrC *** **(AN2358)**	0.15 ± 0.01	0.22 ± 0.02	0.17 ± 0.02	0.00 ± 0.00	0.14 ± 0.02	0.91 ± 0.05	0.28 ± 0.02
***xtrD *** **(AN0250)**	3.95 ± 0.00	34.80 ± 3.70	9.48 ± 2.24	43.12 ± 3.14	15.09 ± 0.03	74.37 ± 1.15	31.12 ± 3.46
***xtrE *** **(AN4148)**	0.18 ± 0.01	0.95 ± 0.15	1.08 ± 0.24	1.16 ± 0.03	0.77 ± 0.00	0.48 ± 0.00	0.45 ± 0.00

Results are presented as mean ± SD.

### Functional characterization of genes encoding putative xylose transporters

**Figure 3:**
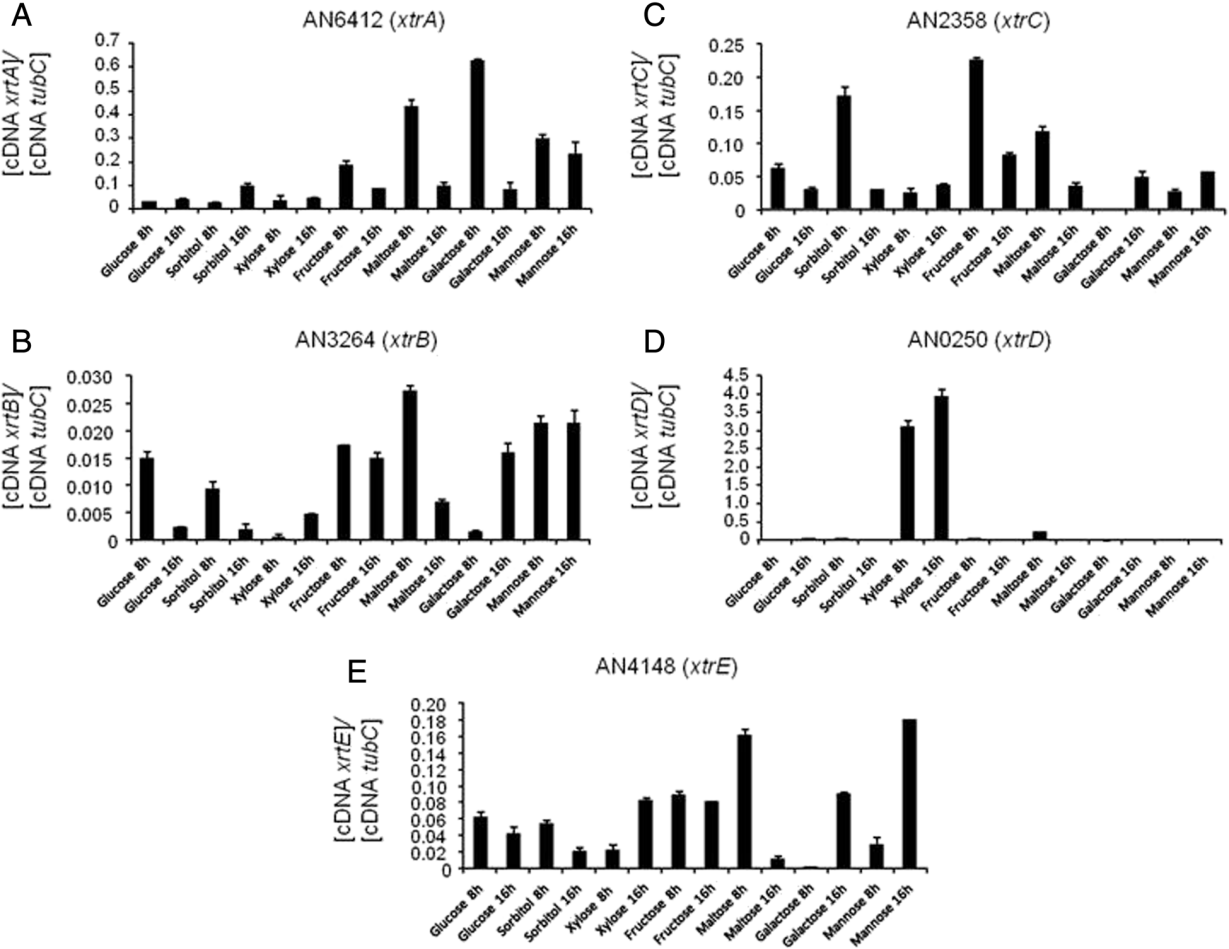
**Expression profiles of five transporter-encoding genes in the presence of various carbon sources.** The *A. nidulans* wild-type strain was grown from conidia for 8 h or 16 h in the presence of glucose 1%, sorbitol 1%, xylose 1%, fructose 1%, maltose 1%, galactose 1% and mannose 1%. Gene expression levels were determined by RT-qPCR for genes AN6412 **(A)** (*xtrA*), AN3264 **(B)** (*xtrB*), AN2358 **(C)** (*xtrC*), AN0250 **(D)** (*xtrD*) and AN4148 **(E)** (*xtrE*). Error bars indicate standard deviation for three biological replicates.

**Figure 4:**
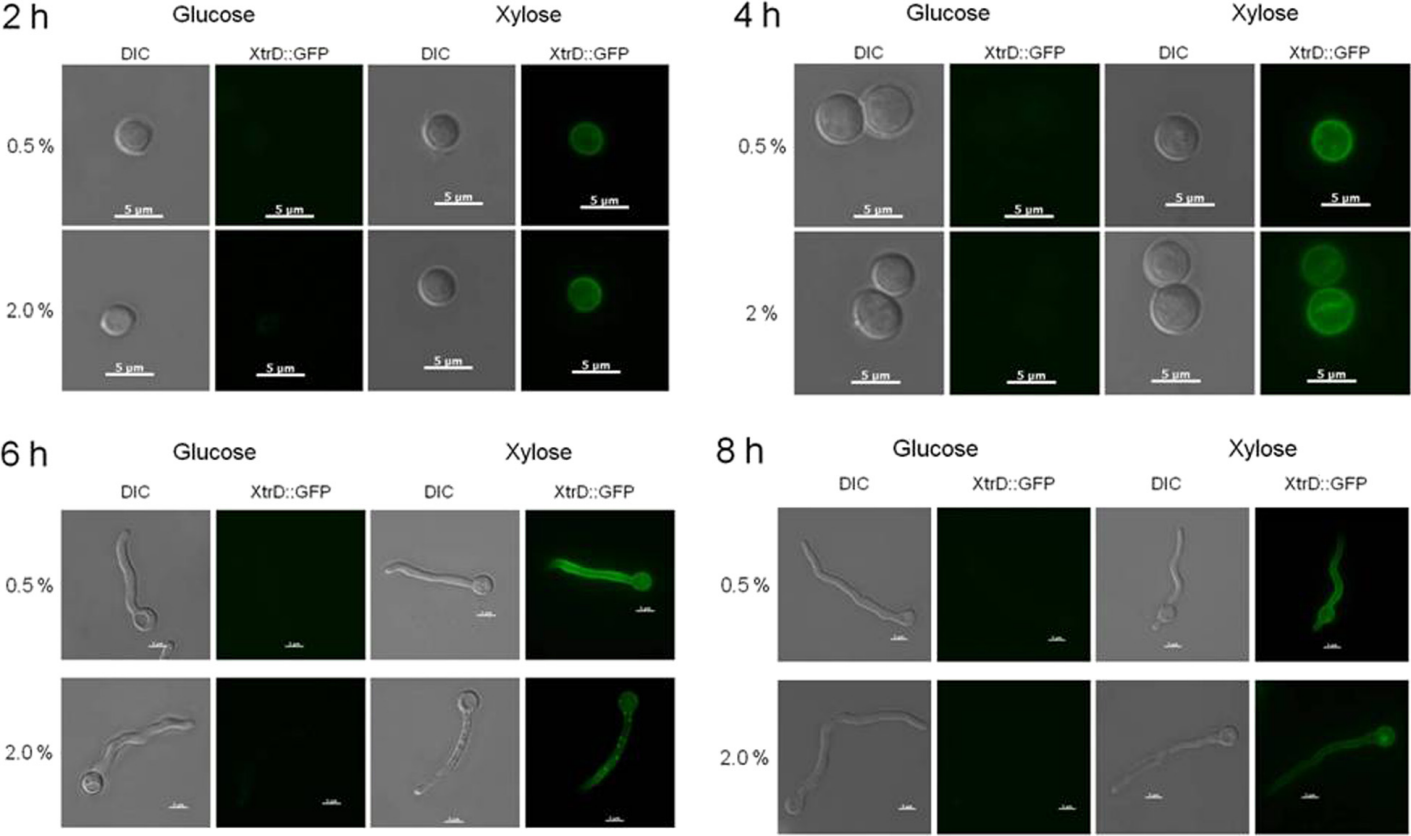
***A. nidulans *XtrD:GFP locates to the cell membrane and in vesicles.** The *A. nidulans* XtrD:GFP strain was grown from conidia in minimal media supplemented with 0.5% and 2% of xylose or glucose for a period of 8 h. Cells were viewed under the microscope at 2-h intervals. Differential interference contrast (DIC) was applied to view unstained cells. Scale bars: 5 μm.

**Figure 5:**
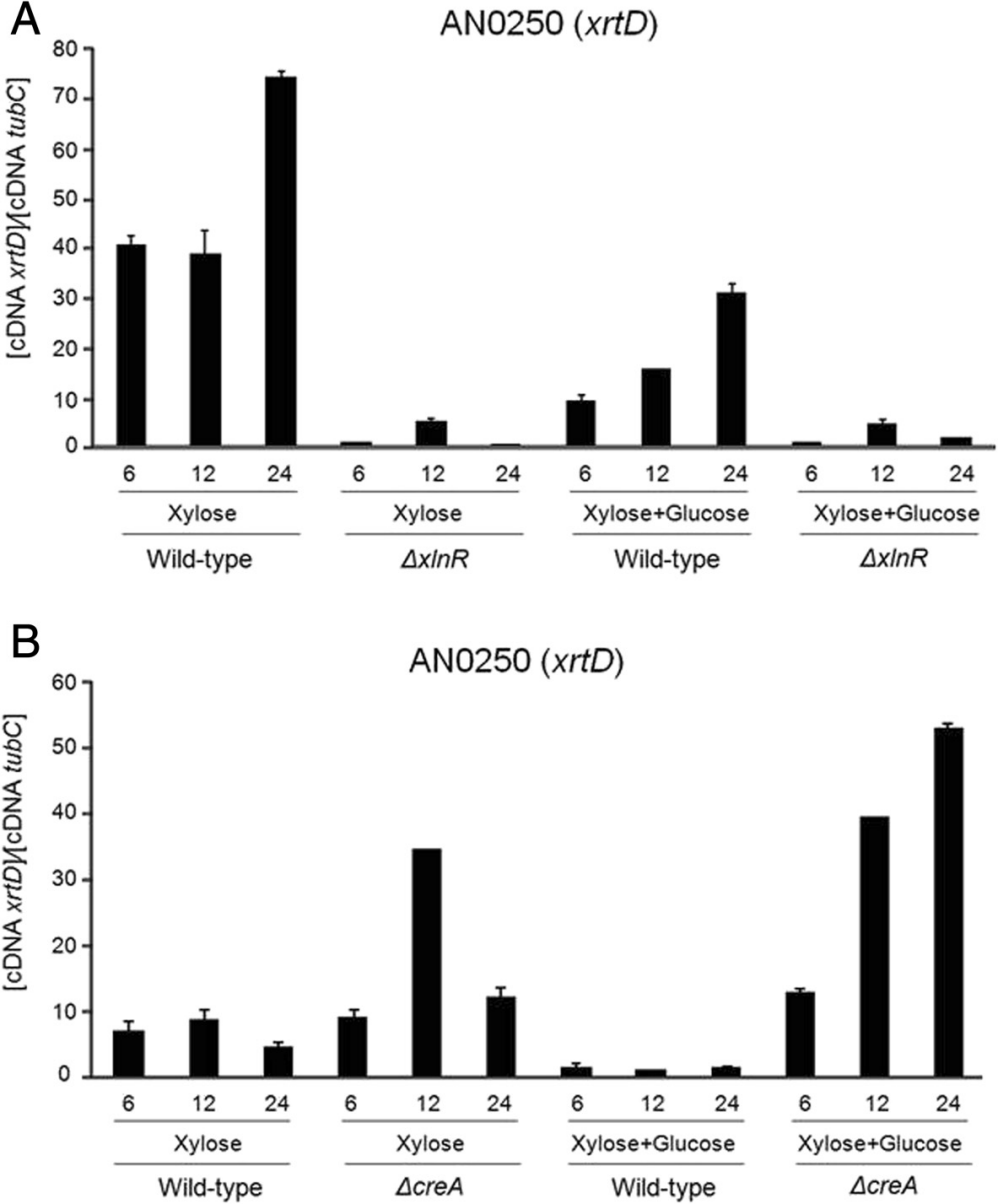
**Transcriptional regulation of *A. nidulans xtrD*, in the presence of xylose, is XlnR and CreA-dependent.** Transcript levels of *xtrD* were determined by RT-qPCR in the *ΔxlnR ***(A)** and *ΔcreA4 ***(B)** strains with comparison to the wild-type strain **(A** and **B)**, grown for 6 h, 12 h and 24 h in the presence of xylose (X) or xylose and glucose (X + G). Error bars indicate the standard deviation for three replicates.

In order to further characterize the five candidate xylose transporters, their expression patterns during growth in the presence of various carbon sources were measured by RT-qPCR (Figure 3). *A. nidulans* was grown from conidia for 8 h or 16 h in media supplemented with glucose, sorbitol, xylose, fructose, maltose or galactose. Genes *xtrA, -B, -C*, and *-E* were induced to a different extent in the presence of all carbon sources. The expression levels of *xtrA*, *xtrB* and *xtrE* increased in the presence of fructose, maltose, galactose and mannose (Figures 3A, 3B and 3E), whereas *xtrC* mRNA levels increased in the presence of sorbitol, fructose and maltose (Figure 3C). The only gene with increased expression levels only in the presence of xylose was *xtrD* (Figure 3D). In order to understand the transcriptional regulation of this gene and the sub-cellular location of the XtrD protein, an XtrD::GFP strain was constructed. All phenotypic traits of this strain were identical to the wild-type strain (data not shown). The XtrD::GFP strain was germinated for 8 h in media containing 0.5% or 2% xylose. A weak fluorescent signal was observed in the XtrD::GFP strain after 1 h in the presence of both 0.5% and 2% xylose. This fluorescence increased considerably after 2 h in the presence of the same xylose concentrations (Figure 4). During the 6 h to 8 h incubation in xylose-rich media (0.5% and 2%), XtrD::GFP was mainly observed at the cell membrane and in a few small spots within the cell (2%), which appear to be vesicles (Figure 4). No fluorescence was observed in glucose-grown cultures at any time point. When incubated in the presence of several other carbon sources, including arabinose and other hexoses, no XtrD::GFP fluorescence was observed (data not shown). This indicates that *xtrD* was transcriptionally induced and translated specifically upon the detection of the pentose sugar xylose. In addition, the expression profile of *xtrD* in the *ΔXlnR* and *ΔcreA4* strains suggests that *xtrD* xylose induction and glucose repression occurred in an XlnR-dependent and partially in a CreA-dependent manner (Figure 5).

**Figure 6:**
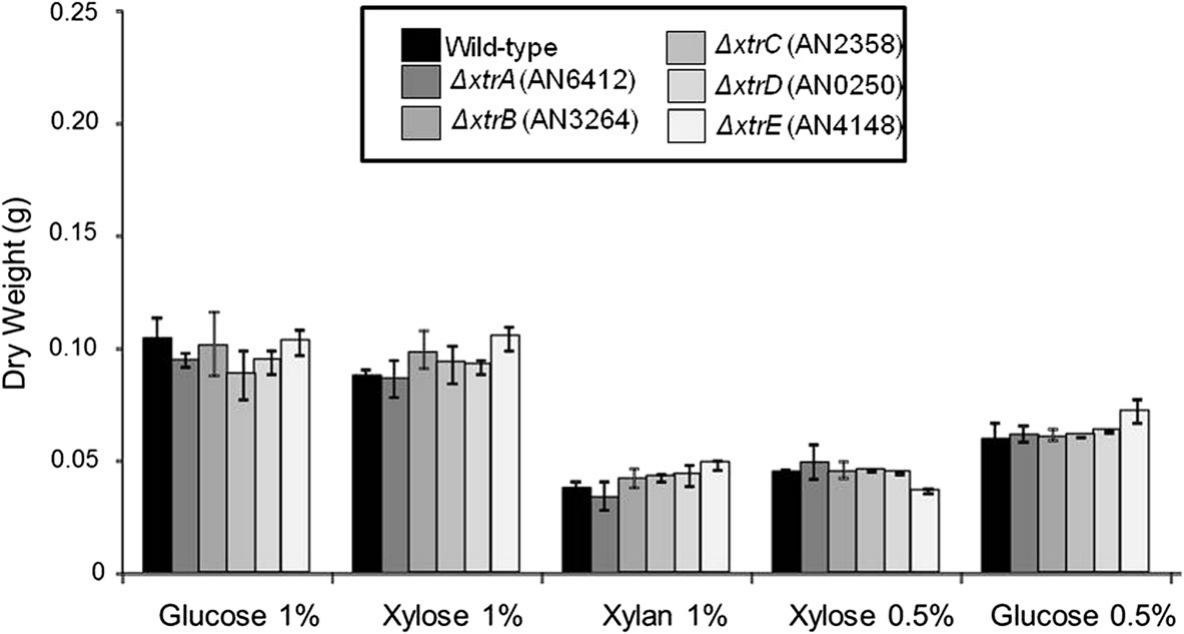
**Growth of *A. nidulans *wild-type and *ΔxtrA-E *mutant strains in the presence of different carbon sources.** Strains were grown from conidia in minimal media supplemented with the relevant carbon source (glucose 1.0%, xylose 1.0%, xylan 1.0%, xylose 0.5% and glucose 0.5%) at 37°C for 24 h. Error bars indicate standard deviation for three biological replicates.

Subsequently, the roles played by the five transporter-encoding genes in carbon source uptake were elucidated by generating *A. nidulans xtrA-E*-null mutants using an *in vivo S. cerevisiae* fusion-based approach (see Methods). Several primary transformed colonies that had homologous integration of *pyrG* at the *xtrA-E* loci (confirmed by PCR) were isolated, and one colony for each gene was selected for further analysis. The growth of the null mutant strains and the wild-type strain (control) was monitored on minimal media agar supplemented with one of the following carbon sources: glucose, xylose, maltose, glycerol, mannose, fructose, acetate, rhamnose, casein, carboxymethylcellulose (CMC), inulin, guar, peptone and pectin at 30°C, 37°C and 44°C. The five deletion strains showed the same growth and conidiation patterns as the wild-type strain under all the tested conditions (data not shown). The growth of the same strains in liquid minimal medium supplemented with either 0.5% or 1.0% glucose, 0.5% or 1.0% xylose and 1.0% xylan was measured through determining fungal dry weight (Figure 6). There was no significant difference in biomass accumulation between the strains in any of the carbon sources (Figure 6). These results showed that the lack of a single transporter, *xtrA-E,* did not result in a loss of growth in the presence of various carbon sources, suggesting gene redundancy.

### Characterization of the xtrD gene in *S. cerevisiae*

**Figure 7:**
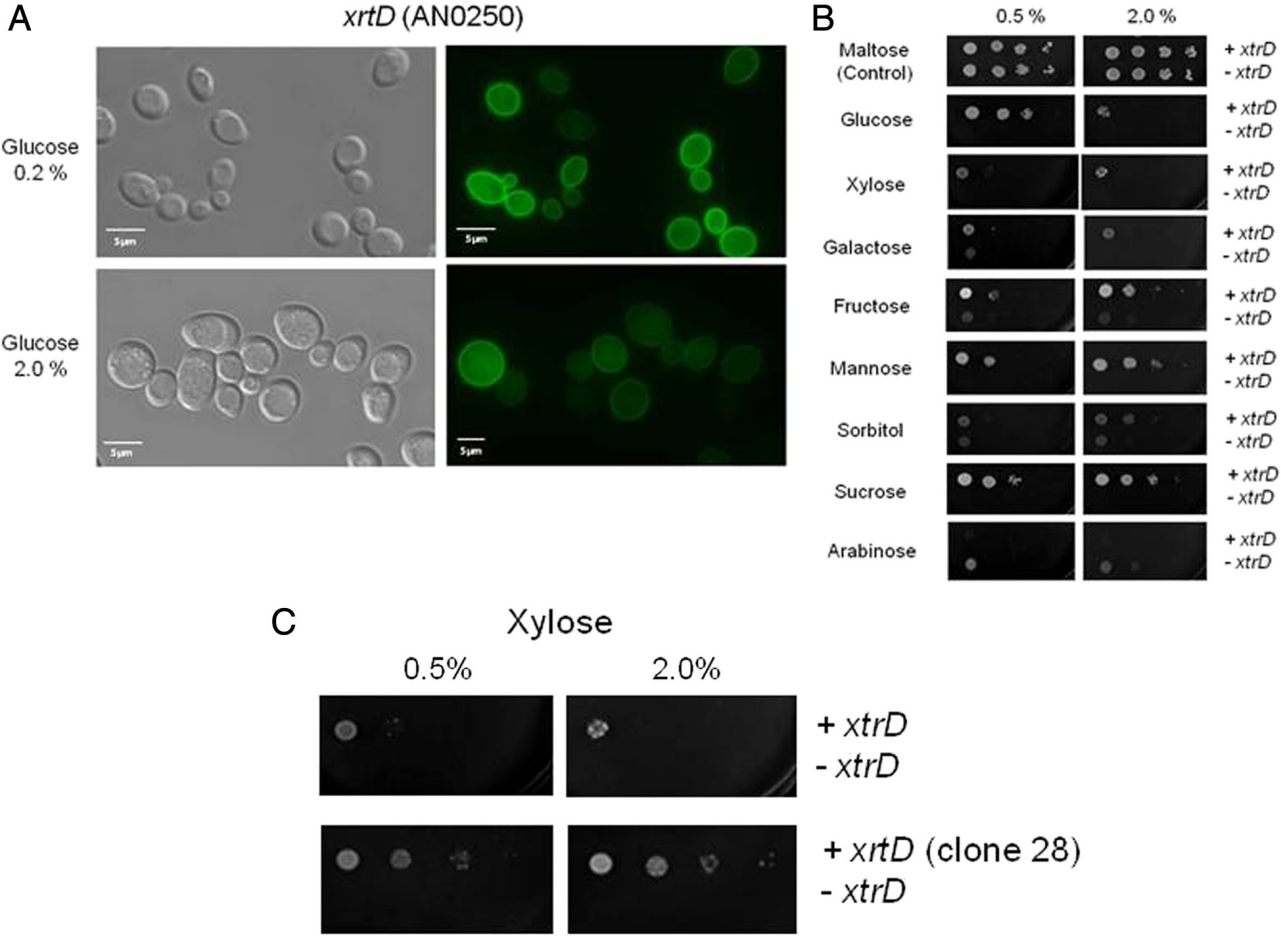
**Comparative growth analysis of yeast cells expressing the *xtrD *transporter gene. (A)** Subcellular localization of XtrD::GFP in *S. cerevisiae* when incubated in glucose-rich media, as determined by fluorescent microscopy (scale bar, 5 μm). **(B)** Growth of strain EBY.VW4000 containing the *xtrD* gene (+ *xtrD*) or harboring the empty expression vector (- *xtrD*) but containing the xylose reduction pathway. Tenfold dilutions were made and cells were spotted onto agar medium supplemented with the relevant carbon source and incubated at 30°C for 144 h. **(C)** Comparative growth of two different *xtrD*-expressing *S. cerevisiae* strains in the presence of xylose. Cells without *xtrD* (- *xtrD*) and cells containing the *xtrD* gene (+ *xtrD*) before and after (clone 28) a 21-day xylose adaptation period were diluted and spotted onto agar plates (as described above).

XtrD was the only transporter specifically induced in the presence of xylose and was therefore selected for further study. Primarily, the correct localization of XtrD in *S. cerevisiae* was confirmed by cloning *xtrD* fused to the *gfp* gene into the centromeric modified vector pRH195, where the construct was under the control of the *HXT7* promoter and terminator regions. Transformed into *S. cerevisiae,* XtrD::GFP was observed to be mainly targeted to the plasma membrane (Figure 7A), justifying the use of *S. cerevisiae* as a model.

The *S. cerevisiae* strain EBY.VW4000 lacks about 20 glucose transporters and is unable to grow on various monosaccharides, including glucose, fructose, mannose, galactose and xylose [21]. *S. cerevisiae* cannot efficiently utilize xylose as a sole carbon source. Therefore, to utilize the EBY.VW4000 as a tool for evaluating the capacity of XtrD to preferentially transport xylose, additional genes encoding enzymes for xylose utilization were introduced. Subsequently genes encoding proteins of the xylose utilization pathway were introduced into EBY.VW4000 via transformation with the pRH274 plasmid containing the *S. stipitis* XR and XDH, plus the *S. cerevisiae* xylulose kinase (*XKS1*) genes (strain EBY + pRH274). These genes were put under the control of the *PGK1*, *ADH1* and *HXT7* constitutive promoters, respectively [9].

The EBY.VW4000 strain possessing the modified xylose utilization pathway was then transformed with pRH195m containing *xtrD* under the control of the *HXT7* constitutive promoter (strain EBY + pRH274 + pRH195m). The modified xylose utilization strain, with and without XtrD were grown in the presence of different monosaccharides. The respective strains were first grown in maltose-rich liquid medium before serial dilutions were prepared and both strains were transferred onto YNB agar plates containing one of the following carbon sources: maltose (control), glucose, xylose, galactose, fructose, mannose, sorbitol, sucrose and arabinose at a concentration of either 0.5% or 2.0%. The drop-out assay showed that the expression of XtrD in the modified xylose utilization EBY + pRH274 + pRH195m strain was able to restore growth of this organism in the presence of glucose, indicating that XtrD was able to transport glucose (Figure 7B). In addition, the expression of XtrD restored the ability to grow on all other carbon sources tested, except for arabinose. This indicates that the transporter encoded by *xtrD* accepts multiple sugars as substrate. Interestingly, the expression of XtrD was only able to partially restore growth in the presence of xylose (Figure 7B).

The EBY + pRH274 + pRH195m strain expressing the components of the xylose utilization pathway and the *xtrD* gene were inoculated onto 10 plates containing minimal media supplemented with 2% xylose. After 21 days of incubation at 30°C, around 40 colonies had grown on the plates. The colonies were then transferred onto new plates containing minimal medium supplemented with 2% xylose. Only four of the colonies (named hereafter clones 16, 22, 28 and 31) displayed consistent growth on xylose (data not shown). A drop-out assay of clone 28 showed improved growth on medium containing xylose, when compared to the growth of the parental *xtrD*-transformed strain, prior to the 3-week adaptation in xylose-rich media (Figure 7C; see also Additional file 2).

To check if any mutations had occurred in the *xtrD* gene, plasmids were extracted from the four clones and the *xtrD* gene was sequenced. Sequencing results confirmed that in these strains no mutations had occurred in the *xtrD* gene (data not shown). Furthermore, to verify that the observed enhanced growth of the clones was not due to any mutations in the genes encoding components of the xylose utilization pathway or in the *xtrD* gene, both plasmids (one containing the three genes required for xylose utilization and one containing *xtrD*) were extracted from all four strains and transformed back into the original EBY.VW4000 strain. Re-transformed strains did not demonstrate enhance growth on xylose when compared to the respective clones, indicating that the acquired mutation(s) responsible for the adaptation to xylose did not reside on these two plasmids and must have had occurred elsewhere in the genome. In support of this hypothesis, both plasmids were cured from the four clones, resulting in a strain that could no longer utilize xylose. The cured clones were then re-transformed first with pRH274 (containing the xylose utilization pathway genes) and subsequently with pRH195 (containing *xtrD)*. Only clones harboring both plasmids were able to grow in the presence of 2% xylose (Additional file 3). These results suggest that we selected for mutation(s) in the EBY + pRH274 + pRH195m strain, beyond the four introduced genes, which enhanced the efficiency of this strain to sustain growth on 2% xylose.

### XtrD has high affinity for xylose

**Figure 8:**
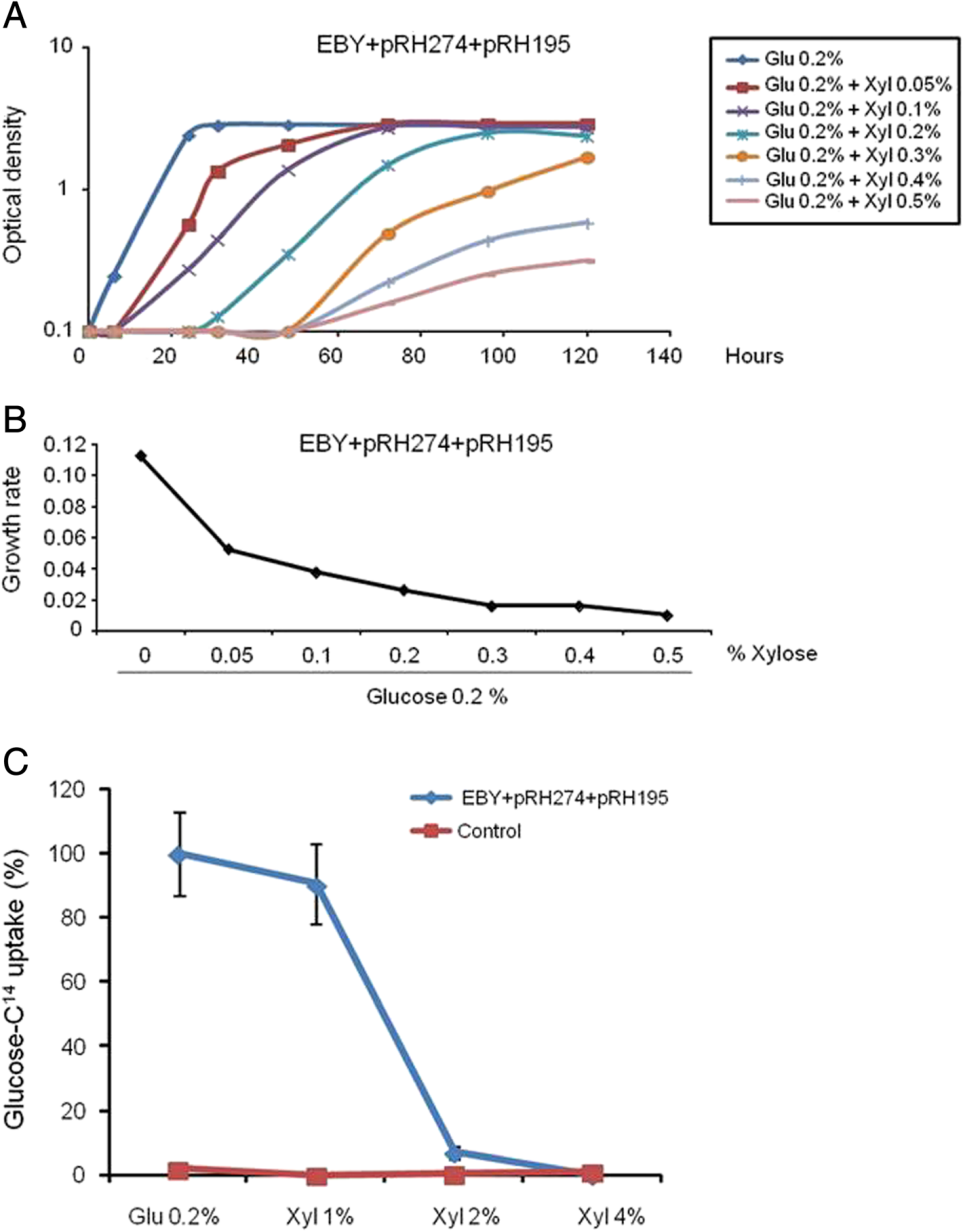
**XtrD has higher affinity for xylose than glucose. (A)***S. cerevisiae* EBY.VW4000 containing the *xrtD* gene or harboring the empty expression vector were grown for 140 h at 30°C in YNB medium plus 0.2% glucose and increasing xylose concentrations. **(B)** Growth rate of the experiments are shown in **(A)**. **(C)** Glucose and xylose substrate specificities of the XtrD transporter. Glucose and xylose specificities of XtrD were determined in yeast cells (strain EBY.VW4000) expressing the respective cDNA. Relative transport levels were determined in the absence of a competitor or in the presence of increasing concentrations of xylose (n = 3, ±, standard deviation). The results are expressed as the percentage of inhibition of the transport with radiolabeled glucose.

The XtrD transporter was able to accept multiple sugars as substrates, such as glucose and xylose (Figure 7B). Considering that XtrD was able to transport glucose (Figure 7B), the original modified xylose utilization EBY.VW4000 strain expressing *xtrD* (non-adapted EBY + pRH274 + pRH195m strain) was grown in the presence of a constant concentration of glucose (0.2%) and an increasing concentration of xylose (0.05 to 0.5%). Figures 8A and B showed that increasing concentrations of xylose dramatically decreased the growth of this strain. To confirm these physiological data, the uptake of (^14^C) glucose in the absence or presence of increasing concentrations of xylose, as a potential transport competitor, was measured (Figure 8C). A 10- and 20-fold excess of unlabeled xylose inhibited the transport of radiolabeled glucose between 90% to 100%, respectively (Figures 8C). In addition, the expression of *xtrD* in *A. nidulans* was higher when grown in the presence of lower concentration of xylose (Additional file 4, and Figure 4 and Figure 8D). These results suggest that XtrD has affinity for xylose, and may be a high-affinity xylose transporter.

## Discussion

Economically viable 2G bioethanol production requires the development of efficient technologies for the co-fermentation of the hexose and pentose sugars stored within plant biomass. The introduction of the xylose utilization pathway from other organisms into *S. cerevisiae* has proven insufficient [7] and xylose transport is proposed to be the limiting step. Filamentous fungi possess a greater capacity to consume pentose sugars [17,18] and represent a possible source of genes for the engineering of pentose utilization in *S. cerevisiae*. Therefore, the aim of this study was to identify xylose transporters from *A. nidulans*, which may enhance xylose consumption in a modified *S. cerevisiae* strain, containing the xylose utilization pathway. Initially, the regulation of xylose utilization in *A. nidulans* was assessed to facilitate the identification of candidate xylose transporters. Xylose uptake was shown to be inhibited by glucose-induced CCR, whereas the carbon catabolite-derepressed strain, *creAd30*, consumed xylose even in the presence of glucose. In agreement, extracellular endoxylanase activity, which is induced by the presence of xylose or xylan, was suppressed by glucose-induced CCR. In contrast, the *creAd30* strain demonstrated increased endoxylanase activity compared to the wild-type strain on xylose and xylose plus glucose-containing media. This confirms that the CreA-mediated CCR inhibits the transcription of genes involved in the utilization of alternative carbon sources when glucose is detected [22,23]. Similar observations have been made in other filamentous fungi such as *Trichoderma reesei*, where glucose inhibits the uptake of the cellulase-inducer sophorose [24].

Genome-wide microarray analyses of *A. nidulans* post transfer to xylose-containing media enabled the identification of several highly upregulated transporter-encoding genes. Five such genes encoded transporters of the MFS. The MFS are single polypeptide secondary carriers that transport small soluble molecules in response to ion gradients [25]. The MFS comprises 17 families of which families 1 (SP - sugar porter family), 5 and 7 are specific for sugar transport [25]. The five candidate transporters demonstrated high sequence similarity (60% to 80% identity at the protein level) to transporters from other fungi, highlighting the conservation of these proteins across species (data not shown). Furthermore, BLASTp analyses indicated that the five candidate transporters were likely to belong to MFS family 1, which in *S. cerevisiae* and filamentous fungi includes members from the SP family that transport hexoses (for example, glucose, galactose), pentoses (for example, xylose), disaccharides (for example, lactose), quinate and inositols [25]. Due to the lack of information concerning the characterization of fungal transporters, it was not possible to predict the primary functions of the five candidate transporters, based on homology to other known transporters. This underlines the importance to conduct detailed investigation into fungal transporters.

Expression profiles of the five transporter-encoding genes were then further characterized in the presence of various monosaccharides including xylose. Only one gene (ID AN0250), named hereafter *xtrD* (*x*ylose *tr*ansporter), was specifically induced in the presence of xylose. This gene and its homologues have already previously been shown to be highly expressed in the presence of xylose in *A. nidulans*, *A. niger* and *A. oryzae*[26]. Furthermore, the *xtrD* homologue in *T. reesei* (ID 50893), was shown to be highly upregulated when transferring the fungus from glucose to a complex lignocellulosic carbon source [27]. Fusion of XtrD with GFP further confirmed the expression of *xtrD* and the location of the corresponding protein to the cell membrane and to vesicles during the first 8 h of incubation of fungal conidia in xylose-rich media.

The regulation of *xtrD* in the presence of repressing and inducing carbon sources was characterized. The induction of *xtrD* was shown to be XlnR-dependent, whereas *xtrD* repression was CreA-dependent, as suggested by the previous phenotypic assays for xylose utilization and endoxylanase activity. Indeed, Andersen *et al*. [26] identified an XlnR binding motif (5′-GGNTAAA-3′) in the promoter region of *xtrD* in *A. nidulans*, *A. niger* and *A. oryzae*. In *Aspergilli*, XlnR is involved in the induction of xylanases, β-xylosidases, cellobiohydrolases, endoglucanases, galactosidases, arabinofuranosidases and carbohydrate esterases in the presence of the inducer D-xylose [28]. In the presence of glucose, the same hydrolases, esterases and transporters are repressed by the CCR, CreA. This allows the fungus to select the most energetically favorable carbon source and not waste energy on the secretion of polysaccharide-degrading enzymes [22,23]. Both transcription factors, XlnR and CreA, therefore, coordinate the regulation of genes encoding proteins required for carbon source metabolism and acquisition, including transporters.

The genome of *A. nidulans* contains over 30 genes encoding transporters of the MFS (http://www.aspgd.org), suggesting the existence of functional redundancy among transporters. Single-gene disruption studies of the five candidate xylose transporter-encoding genes had no significant impact on fungal growth on different carbon sources including xylose, probably due to redundancy. An alternative strategy was required to assess gene function. Subsequently, the most promising candidate, *xtrD*, was cloned into the *S. cerevisiae* strain EBY.VW4000 that lacks all hexose transporters, harbors the components of the xylose reductive pathway, but is unable to grow on a range of sugars including glucose and xylose. The correct sub-cellular localization of XtrD::GFP to the plasma membrane was confirmed. The introduction of *xtrD* restored the ability to grow on glucose, fructose, mannose, sorbitol and sucrose. Therefore, XtrD can transport multiple sugars, which is not surprising as many fungal transporters exhibit a wide range of substrate binding [11]. This is advantageous when fungi encounter complex lignocellulosic material, which contains multiple hexose and pentose sugars. However, the recovery of growth on xylose was limited, indicating that XtrD and components of the xylose utilization pathway are not enough to fully restore growth on this pentose sugar. Interestingly, when expressed in *S. cerevisiae*, XtrD showed a high affinity for xylose, even allowing the growth inhibition of *S. cerevisiae* in the presence of glucose.

To further enhance xylose utilization, the *xtrD*-containing, transformed strain was incubated for three weeks on xylose-rich media, resulting in cells that exhibited much improved growth on xylose via adaptive evolution. Attempts made to identify the cause of this improvement, revealed that this strain required both plasmids (harboring the xylose utilization genes and transporter-encoding gene) for growth on xylose, but these plasmids alone did not confer the enhanced ability to grow on xylose. Therefore, the mutation(s) that occurred within the *S. cerevisiae* genome had a synergistic effect, along with *xtrD* and the xylose reduction pathway, on xylose utilization. A similar investigation by Saloheimo *et al.*[29], where *S. cerevisiae* was transformed with the *T. reesei* gene *xlt1* (ID 104072), produced similar results and conclusions. Adaptive evolution is a powerful tool for improving the growth of yeast strains in different carbon sources, but pinning down the resulting mutations responsible for the observed phenotype requires whole genome sequencing and subsequent genetic mutation studies, which is labor-intensive and costly. An alternative method to adaptive evolution in yeast could be targeted protein engineering. Young *et al*. [30] used directed evolution in yeast to create mutations in several transporters. They managed to engineer transporters with improved xylose-transporting capacity and although challenges remain such as the tradeoff between transport efficiency and substrate affinity, or the preference of glucose to xylose, protein engineering seems to be a promising tool to create ideal xylose transporters for lignocellulose biomass conversion.

## Conclusion

Efficient pentose uptake and the subsequent fermentation remains a bottleneck in 2G bioethanol production. Screening of the genomes of filamentous fungi (for example, *A. nidulans*, *T. reesei*) has resulted in the identification of promising xylose transporters, but these proteins probably need to be fine-tuned in order to improve their efficiency and capacity for pentose uptake and to de-select for the preference towards glucose. This study characterized the regulation and substrate specificity of a transporter that represents a good candidate for further directed mutagenesis. Investigation into the area of sugar transport (characterization of transporters from the MFS and directed mutagenesis of transporters) in fungi presents a crucial step for improving the conversion of lignocellulosic biomass into bioethanol. Nonetheless, the localization of the adaptive mutations beyond the introduced xylose utilization genes demonstrates the complexity of the system that involves many additional genes, providing substantial room for significant biotechnological improvements in xylose utilization efficiencies.

## Methods

### Strains, media and culture methods

The *A. nidulans* and *S. cerevisiae* strains used in this study are described in Table 5. All *A. nidulans* strains were grown in minimal media (1% (w/v) glucose, 50 ml of a 20 × salt solution (120 g/l NaNO_3_, 10.4 g/l KCl, 30 g/l KH_2_PO_4_, 10.4 g/l MgSO_4_), 1 ml of 5 × trace elements (22.0 g/l ZnSO_4_, 11 g/l boric acid, 5 g/l MnCl_2_, 5 g/l FeSO_4_, 1.6 g/l CoCl_2_, 1.6 g/l CuSO_4_, 1.1 g/l (NH_4_)_2_MoO_4_, 50 g/l ethylenediaminetetraacetic acid (EDTA)) and adjusted to pH 6.5 with NaOH. *S. cerevisiae* was grown in a solution containing 7 g/l Yeast Nitrogen Base without amino acids, histidine (0.1 g/l), lysine (0.1 g/l), leucine (0.1 g/l), tryptophan (0.1 g/l), uridine (1.2 g/l) and uracil (1.2 g/l). All reagents were obtained from Sigma Aldrich (St. Louis, MO, USA), except where stated.

**Table 5:** ***A. nidulans *** **and *S. cerevisiae *** **strains used in this work**

**Strain**	**Genotype**	**References**
*A. nidulans*		
TNO2a3	*pyrG89;pyroA4; Δnku70::argB*	Reference [31]
R21	*yA1 pabaA1*	Reference [32]
*ΔxlnR*	*pabaA1;FwA1;uaY9;pyrG89;ΔxlnR::pyr4*	Reference [33]
*ΔcreA4*	*pantoB100;creΔ4;ua211*	Reference [34]
*creAd30*	*creA30; biA1; wA3*	Reference [35]
ΔAN0250	*pyrG89; pyroA4; Δnku70::argB ΔxtrD::pyrG*	This work
ΔAN6412	*pyrG89; pyroA4; Δnku70::argB; ΔxtrA::pyrG*	This work
ΔAN3264	*pyrG89; pyroA4; Δnku70::argB; ΔxtrB::pyrG*	This work
ΔAN2358	*pyrG89; pyroA4; Δnku70::argB; ΔxtrC::pyrG*	This work
ΔAN4148	*pyrG89; pyroA4; Δnku70::argB; xtrE::pyrG*	This work
XtrD::GFP	*pyrG89; pyroA4; Δnku70::argB; XtrD::GFP::pyrG*	This work
*S. cerevisiae*		
EBYVW4000	*CEN.PK2-1C hxt13Δ::loxP; hxt15ΔloxP; hxt16Δ::loxP; hxt14Δ::loxP; hxt12Δ::loxP; hxt9Δ::loxP; hxt11Δ::loxP; hxt8Δ::loxP; hx10Δ::loxP; hxt514Δ::loxP; hxt2Δ::loxP; hxt367Δ::loxP; gal2Δ stl1Δ::loxP; agt1s::loxP; ydl247wΔ::loxP; yjr160Δ::loxP*	Reference [21]
EBI + XrtD:: GFP	EBYVW4000 pRH195 XtrD::GFP	This work
EBI + pRH274 + pRH195m	EBYVW4000 pRH195 *xtrD* pRH274	This work
EBI + pRH195	EBYVW4000 pRH195	This work
EBI + pRH274 + pRH195	EBYVW4000 pRH195 pRH274	This work
Clone 16	EBYVW4000 pRH195m pRH274 mutated	This work
Clone 22	EBYVW4000 pRH195m pRH274 mutated	This work
Clone 28	EBYVW4000 pRH195m pRH274 mutated	This work
Clone 31	EBYVW4000 pRH195m pRH274 mutated	This work

### Endoxylanase activity

Endoxylanase (endo-1,4-β-xylanase) activity was measured, using Azo-Xylan from Birchwood (Megazyme International, Bray, Ireland) as a substrate. The enzyme assay was carried out according to the manufacturer’s (Megazyme) instructions. Briefly, 500 μl of the sample supernatant (containing the xylanases) was mixed with 500 μl of 100 mM sodium acetate buffer (pH 4.5). Then, 500 μl of the diluted enzyme preparation was mixed with 500 μl substrate solution (1% w/v Azo-Xylan). The samples were incubated at 40°C for 10 minutes and then the reactions were stopped via the addition of 2.5 ml ethanol (95% v/v). Samples were centrifuged for 10 minutes at 1,000 × *g*. The supernatant was removed and absorbance was measured at 590 nm. Enzymatic activity was determined using the Mega-Calc™ software (Megazyme International). One unit of enzymatic activity was defined as the amount of enzyme required to release 1 mM of D-xylose from arabinoxylan per minute (pH 4.5) at 40°C.

### Xylose and glucose uptake

*A. nidulans* strains were grown in 50 ml minimal media supplemented with 1% (w/v) glucose or 1% (w/v) xylose at 37°C, 150 rpm, for different time periods. At each time point, 5.0 ml of the culture supernatant was harvested. Glucose and xylose concentrations of the supernatants were measured using the Glucose GOD-PAP Liquid Stable Mono-reagent enzymatic kit from LaborLab Laboratories Ltd. (Guarulhos, SP, Brazil) and the D-xylose assay kit from Megazyme. All assays were carried out according to manufacturer’s instructions.

### Molecular techniques

Standard *A. nidulans* strain construction and transformation techniques were used throughout this study [36]. All DNA manipulations were performed as previously described [37]. Deletion cassettes were provided by the Fungal Genetics Stock Centre (FGSC) (http://www.fgsc.net). PCR reactions were performed using the TaKaRa Ex Taq DNA Polymerase (Takara BIO Inc., Shiga, Japan), according to manufacturer’s instructions. Primer-pair details are described in Additional file 5. Transformed strains were grown on minimal medium depleted in uridine and uracil and checked for the presence of the correct insertion by PCR.

### Cloning of heterologous genes

Gene fragments were amplified by PCR, using Phusion DNA polymerase (Thermo Fisher Scientific, Waltham, MA, USA) according to manufacturer’s protocols. PCR primers used for all gene amplifications and plasmids are listed in Additional files 5 and 6. Plasmid pRH195 was linearized using restriction enzymes *Sa*lI and *Spe*I [New England Biolabs (NEB), Ipswich, MA, USA) and yeast were transformed with plasmid pRH195 and the appropriate DNA fragments according to the previously described protocol [38].

### Yeast growth rates

The growth rates of the transformed yeast strains when grown in SC-URA for 24 h at 30°C, 150 rpm, were performed in triplicate and recorded via measuring the optical density (OD) of the cultures at 640 nm. Subsequently, these cells were transferred to 100 ml YNB media supplemented with the respective carbon source at 30°C, 150 rpm, at a starting OD_640_ of 0.1.

### *A. nidulans* growth rates

*A. nidulans* strains were cultivated (from 10^6^ conidia) in 25 ml minimal media supplemented with the respective carbon source at 37°C, 150 rpm, for different periods of time. At each time point, mycelia were harvested, snap-frozen in liquid nitrogen and freeze-dried, before the dry weight was recorded.

### Staining and microscopy

Conidia of the *A. nidulans xtrD*::GFP strain were inoculated onto coverslips mounted within 5 ml of minimal media supplemented with the respective carbon source, for the specified conditions (see Results). Germinating conidia were then fixed (3.7% formaldehyde, 50 mm sodium phosphate buffer pH 7.0, 0.2% Triton X-100) for 30 minutes at room temperature. Coverslips were then rinsed with PBS buffer (140 mM NaCl, 2 mM KCl, 10 mM NaHPO_4_, 1.8 mM KH_2_PO_4_, pH 7.4) and incubated for 5 minutes at room temperature in a solution containing 100 ng/ml 4′,6-diamidino-2-phenylindole (DAPI; Sigma Chemical, St. Louis, MO, USA). Coverslips were again washed with PBS buffer at room temperature and examined under the microscope.

Mycelia were viewed under a Carl Zeiss (Jena, Germany) AxioObserver.Z1 fluorescent microscope equipped with a 100-W HBO mercury lamp, using the 100× magnification oil immersion objective (EC Plan-Neofluar, NA 1.3). Phase-contrast brightfield and fluorescent images were taken with an AxioCam camera (Carl Zeiss) and processed using AxioVision software (version 3.1). Images were exported and further processed using Adobe Photoshop 7.0 (Adobe Systems Incorporated, San Jose, CA, USA).

### RNA extraction, cDNA synthesis and real-time PCR reactions

To quantify gene expression levels in the presence of different carbon sources, a total of 10^7^*A. nidulans* conidia were inoculated in minimal media supplemented with the respective carbon source for 8 h or 16 h at 37°C, 150 rpm. Mycelia were harvested and snap-frozen in liquid nitrogen. For media transfer experiments, cultures grown in the presence of fructose (control) were inoculated with 10^7^ wild-type *A. nidulans* conidia were inoculated in minimal medium supplemented with 25 mM fructose for 16 h at 37°C, 150 rpm. Mycelia were then washed three times with sterile water and transferred into minimal medium containing 1% xylose or 1% xylose plus 1% glucose, for 6 h, 12 h and 24 h. All mycelia were harvested and snap-frozen in liquid nitrogen.

Mycelia were then ground into a fine powder under liquid nitrogen and RNA was extracted using Trizol (Invitrogen, Carlsbad, CA, USA), according to manufacturer’s instructions. The quality of the RNA (10 μg) was checked on a 1.2% agarose gel containing 2.2 M formaldehyde. RNA samples were DNAse-treated as previously described [39], purified with the RNeasy^®^ Mini Kit (Qiagen, Valencia, CA, USA) and quantified using the NanoDrop^®^ 2000 Thermo Scientific (Uniscience, São Paulo, SP, Brazil) machine.

The cDNA was synthesized from 20 μg RNA (Superscript II kit, Invitrogen), according to manufacturer’s instructions. All RT-qPCR reactions were performed using the ABI 7500 Fast Real-Time PCR System (Applied Biosystems, Foster City, CA, USA) and the SYBR Green PCR Master Mix kit (Applied Biosystems), according to manufacturer’s instructions. Reactions and calculations were performed as previously described [39]. Primer pairs are listed in Additional file 5.

### Microarray slides construction and gene expression methods

Microarray slides were designed exactly as described previously [40]. Samples were labeled with Cy-3 or Cy-5-dUTP using the two-color microarray-based gene expression analysis kit (Quick Amp Labeling Kit, Agilent Technologies™, Santa Clara, CA, USA), according to the manufacturer’s instructions. Amplification and labeling of cRNA were performed through the addition of the Agilent™ Transcription Master Mix (20 μl 4X Transcription Buffer, 6 μl 0.1 M DTT, 8 μl NTP mix, 6.4 μl 50% PEG, 0.5 μl RNase OUT, 0.6 μl inorganic pyrophosphatase, 0.8 μl T7 RNA Polymerase, 2.4 μl Cyanine 3-CTP for the control samples, or cyanine 5-CTP for the treated samples, and 15.3 μl nuclease-free water). Samples were then incubated at 40°C for 2 h. The labeled cRNA was purified using the RNeasy^®^ Mini Kit (Qiagen) and quantified on the NanoDrop^®^ 2000 Thermo Scientific (Uniscience).

To prepare samples for hybridization, 825 ng of each labeled cRNA was mixed with Agilent™ (Agilent^TM^ Technologies) Fragmentation Mix (11 μl 10X blocking agent, 2.2 μl 25X fragmentation buffer, and nuclease-free water to bring the volume to 52.8 μl) and incubated at 60°C for 30 minutes. The fragmentation reactions were stopped through the addition of 55 μl of 2X GE Hybridization Buffer HI-RPM. For microarray hybridizations, 100 μl of the sample was added to the microarray slide, which was then mounted in an Agilent™ Microarray Hybridization Chamber, within an Agilent G2545A hybridization oven for 17 h at 65°C. After hybridization, microarray slides were washed according to the manufacturer’s instructions and scanned using the GenePix^®^ 4000B microarray scanner (Molecular Devices, Sunnyvale, CA, USA).

### Gene expression analysis

The extraction of data from the TIFF files, generated through scanning the microarray slides, was performed using the Agilent Feature Extraction (FE) Software version 9.5.3.1 (Agilent Technologies). This software used the linear Lowess algorithm to subtract the background noise and obtain normalized intensity values. Normalized values were uploaded into the software Express Converter (version 2.1, TM4 platform available at http://www.tm4.org/utilities.html), which converts the Agilent file format to the multi-experiment viewer (mev) file format that is compatible with the TM4 software used for microarray analysis (available at http://www.tm4.org/). The mev files were uploaded into the MIDAS software (TM4 platform), where averages for each gene-replicate, from the biological replicates, were generated. Finally, analysis of the mev files was carried out using the TIGR MeV (TM4 platform, Multi Experiment Viewer, available at http://www.tigr.org/software/microarray.shtml) software. Differentially expressed genes were defined as those that had a mean log_2_ expression ratio statistically different from 0, identified through applying the one-class *t-*test (*P* > 0.01).

### Competition assays

For competition assays using *S. cerevisiae* strains, 500 ml of SC-Trp medium supplemented with 0.2% glucose (11 mM) were inoculated with the EBY.WV4000 strain harboring the *xtrD* gene. Cultures started with an initial OD_640_ of 0.1 and were grown until they reach OD_640_ of approximately 0.6. Cells were harvested by centrifugation (4,000 rpm), washed twice with 50 ml ice-cold water and re-suspended in 1,250 μl of water. A total of 400 μl of these cells was diluted in 800 μl of water and 40-μl aliquots were transferred to 15 ml falcon tubes which were then incubated at 30°C for 5 minutes for temperature equilibration. For competition experiments, 10 μl of water containing different concentrations of xylose (competitor carbon source) plus 0.2 μCi of ^14^C-glucose were added. Sugar uptake was allowed for 5 seconds through vigorous vortexing. The reaction was then immediately stopped by quenching with 1.5 ml ice-cold water and filtration over nitrocellulose filters (Fisher Scientific, Waltham, MA, USA) mounted in a vacuum manifold, followed by two consecutive washes with 1.5 mL of ice-cold water. Filters were then transferred to 8 ml of ScintiSafeTM Econo1 scintillation liquid (Fisher Scientific). The D-(U-14C) glucose taken up by cells was measured using the Tri-Carb^®^ 2100TR Liquid Scintillation Counter (Perkin Elmer, Waltham, MA, USA).

## Abbreviations

1G: first-generation technologies; 2G: second generation technologies; CCR: carbon catabolite repression; mev: multi-experiment viewer; MFS: major facilitator superfamily; NAD: nicotinamide adenine dinucleotide; NADH: reduced nicotinamide-adenine dinucleotide; NADPH: Nicotinamide adenine dinucleotide phosphate; OD: optical density; PBS: phosphate-buffered saline; XDH: xylitol dehydrogenase; XKS1: *S. cerevisiae* xylulose kinase; XR: xylose reductase.

## Competing interests

The authors declare that they have no competing interests.

## Authors’ contributions

LNAR, NAB, TFR, MHS, and FR: conception and design, data collection and analysis, manuscript writing and final approval of the manuscript. ACC, MS and JFM: data collection and analysis, critical revision and final approval of the manuscript. GHG: conception and design, financial support, manuscript writing, final approval of manuscript. All authors read and approved the final manuscript.

## Supplementary Material

Additional file 1 List of genes expressed upon growth on xylose 1%. Click here for file

Additional file 2 **Growth curves for the *S. cerevisiae *clone 28.** (A) Xylose 0.1%; (B) xylose 0.2%; and (C) Xylose 1%. Click here for file

Additional file 3 **Cured cells transformed or not with pRH195m + *xtrD *** **and pRH274.** Click here for file

Additional file 4 **The qPCR for *xtrD *** **upon *A. nidulans *** **growth on different concentrations of glucose or xylose.** Click here for file

Additional file 5 Primers used in this work. Click here for file

Additional file 6 Plasmids used in this work. Click here for file
