# Supplementary material for: Functional characterization of a xylose transporter in Aspergillus nidulans
Source: Biotechnol Biofuels. 2014 Apr 1;7:46. doi: 10.1186/1754-6834-7-46 (PMC4021826; doi:10.1186/1754-6834-7-46)
Supplement: Additional file 2 — Growth curves for the S. cerevisiae clone 28. (A) Xylose 0.1%; (B) xylose 0.2%; and (C) Xylose 1%. [file 1754-6834-7-46-S2.pptx]

## Slide 1
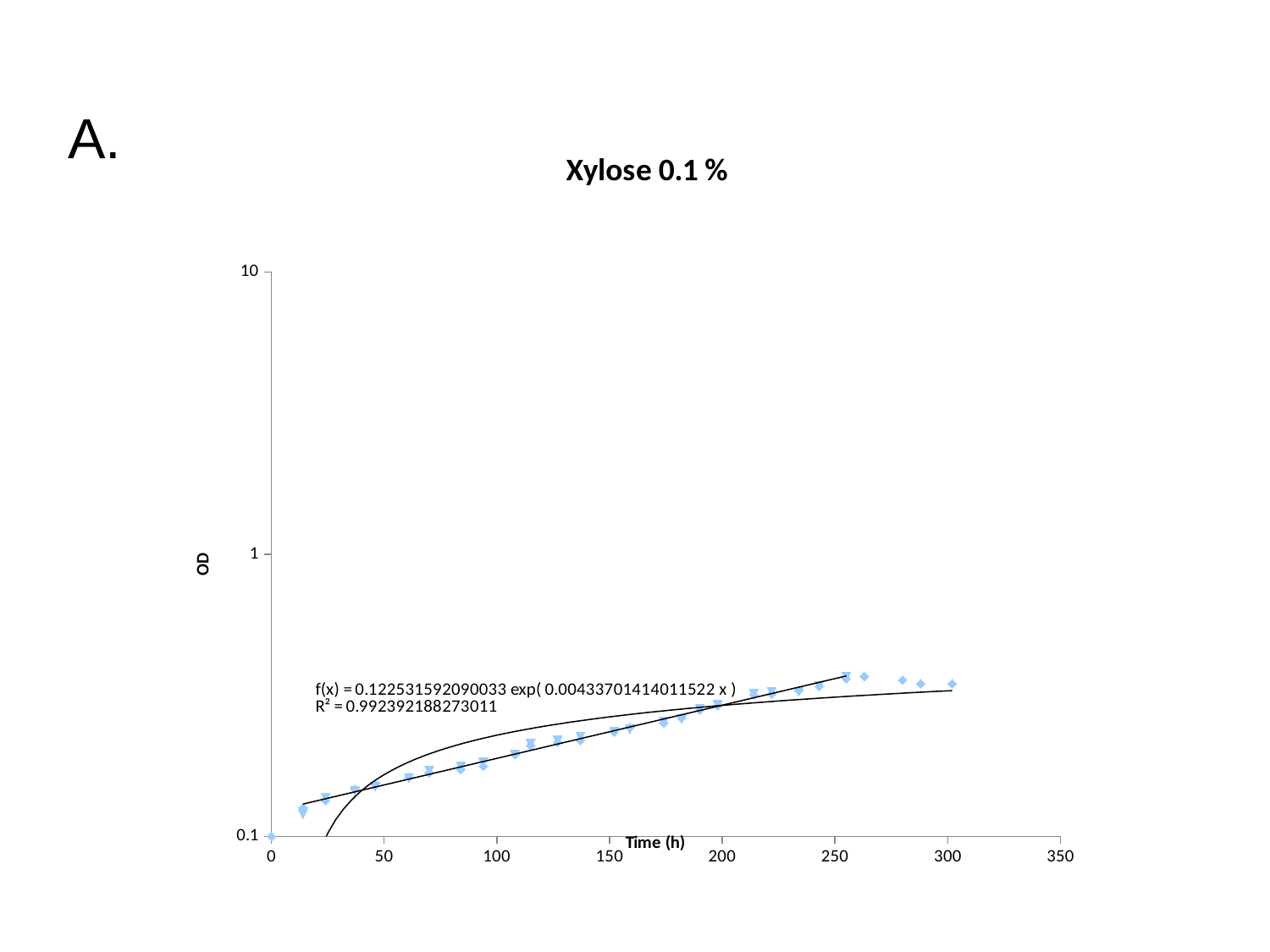

A.
### Chart: Xylose 0.1 %
| Category | | |
|---|---|---|

## Slide 2
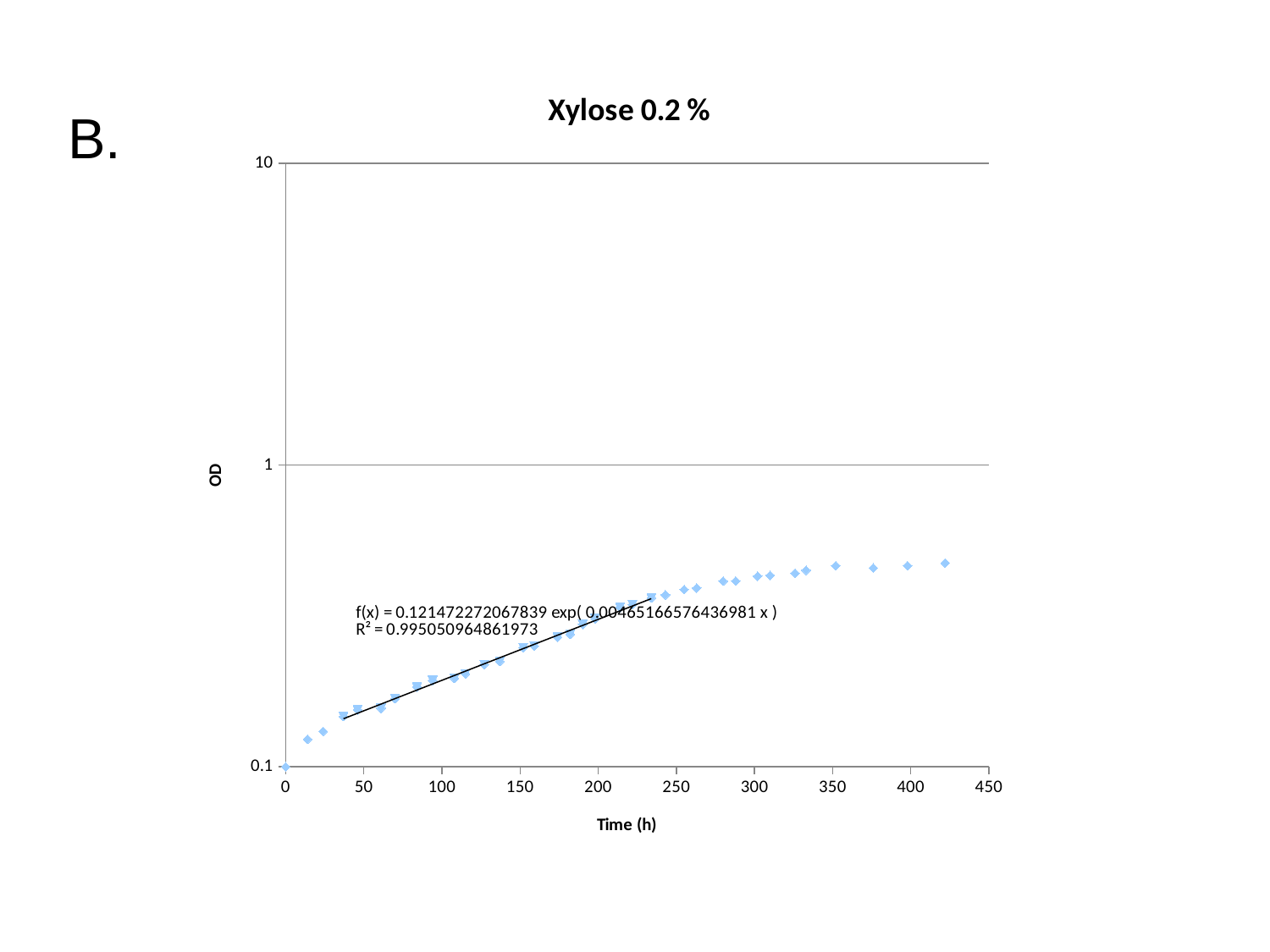

### Chart: Xylose 0.2 %
| Category | | |
|---|---|---|B.

## Slide 3
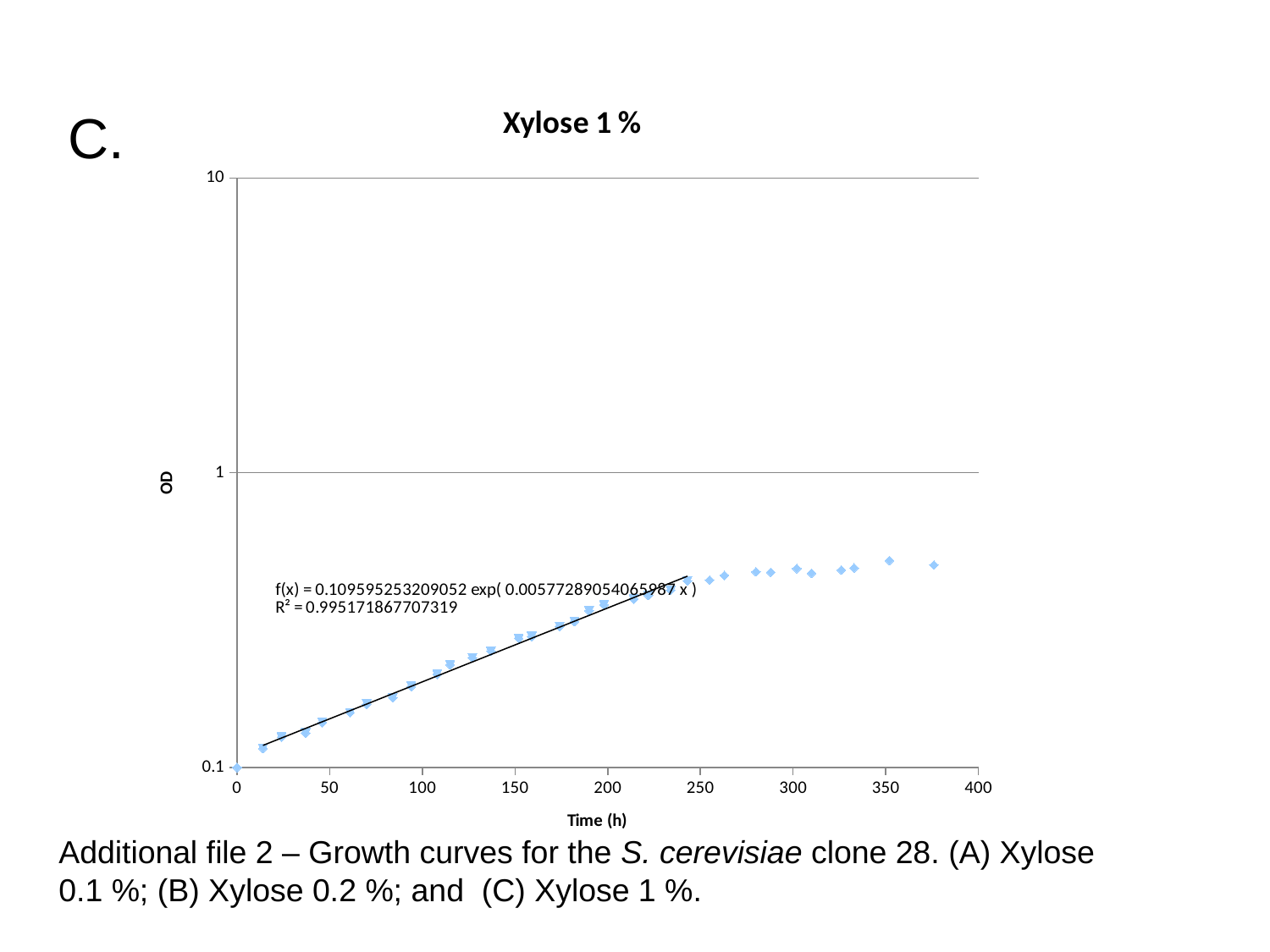

### Chart: Xylose 1 %
| Category | | |
|---|---|---|C.
Additional file 2 – Growth curves for the S. cerevisiae clone 28. (A) Xylose
0.1 %; (B) Xylose 0.2 %; and (C) Xylose 1 %.
