# Supplementary material for: Functional characterization of a xylose transporter in Aspergillus nidulans
Source: Biotechnol Biofuels. 2014 Apr 1;7:46. doi: 10.1186/1754-6834-7-46 (PMC4021826; doi:10.1186/1754-6834-7-46)
Supplement: Additional file 3 — Cured cells transformed or not with pRH195m + xtrD and pRH274. [file 1754-6834-7-46-S3.pptx]

## Slide 1
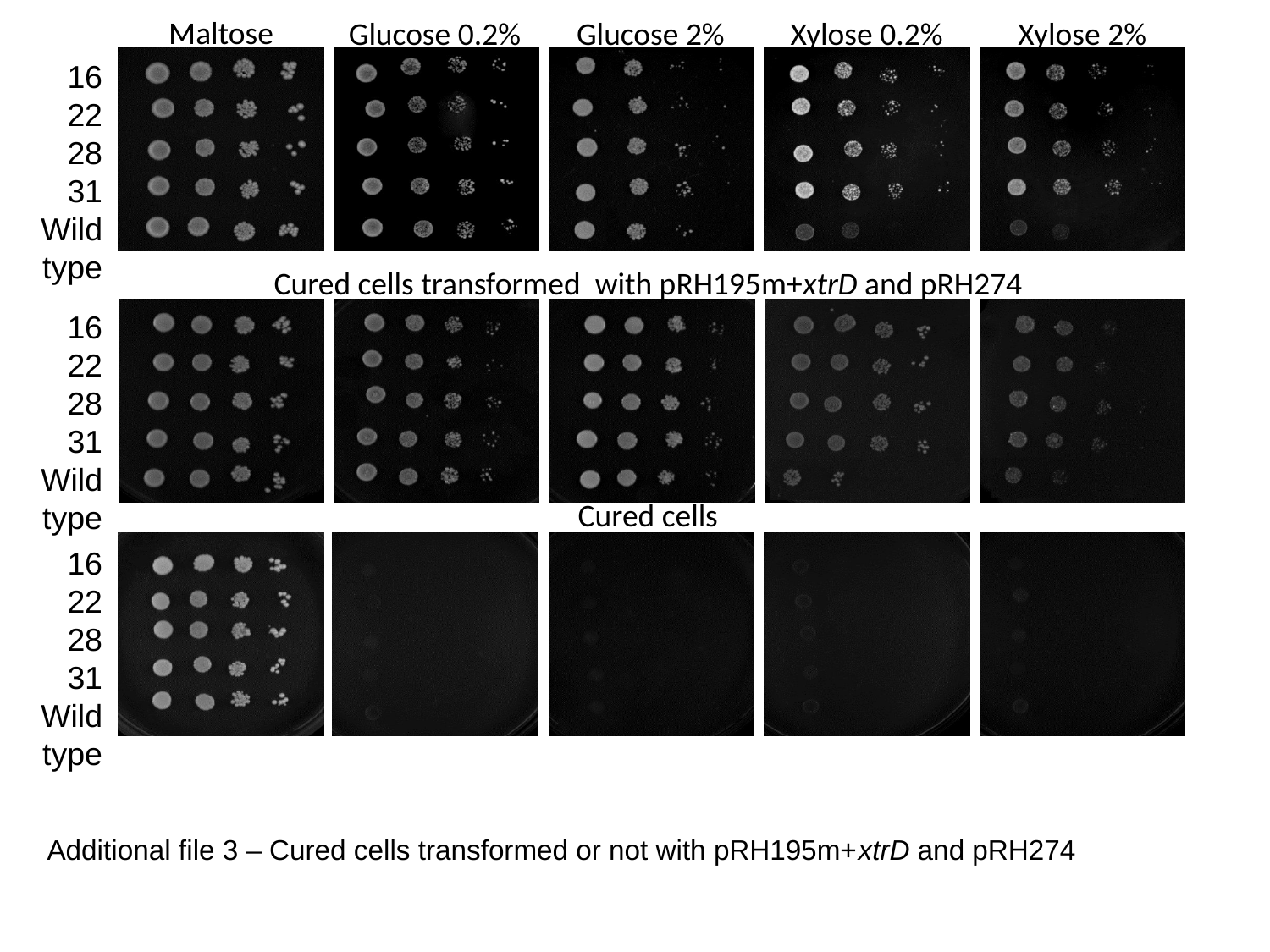

Maltose
Glucose 0.2%
Glucose 2%
Xylose 0.2%
Xylose 2%
16
22
28
31
Wild type
Cured cells transformed with pRH195m+xtrD and pRH274
16
22
28
31
Wildtype
Cured cells
16
22
28
31
Wildtype
Additional file 3 – Cured cells transformed or not with pRH195m+xtrD and pRH274
