# Supplementary material for: Functional characterization of a xylose transporter in Aspergillus nidulans
Source: Biotechnol Biofuels. 2014 Apr 1;7:46. doi: 10.1186/1754-6834-7-46 (PMC4021826; doi:10.1186/1754-6834-7-46)
Supplement: Additional file 4 — The qPCR for xtrD upon A. nidulans growth on different concentrations of glucose or xylose. [file 1754-6834-7-46-S4.pptx]

## Slide 1
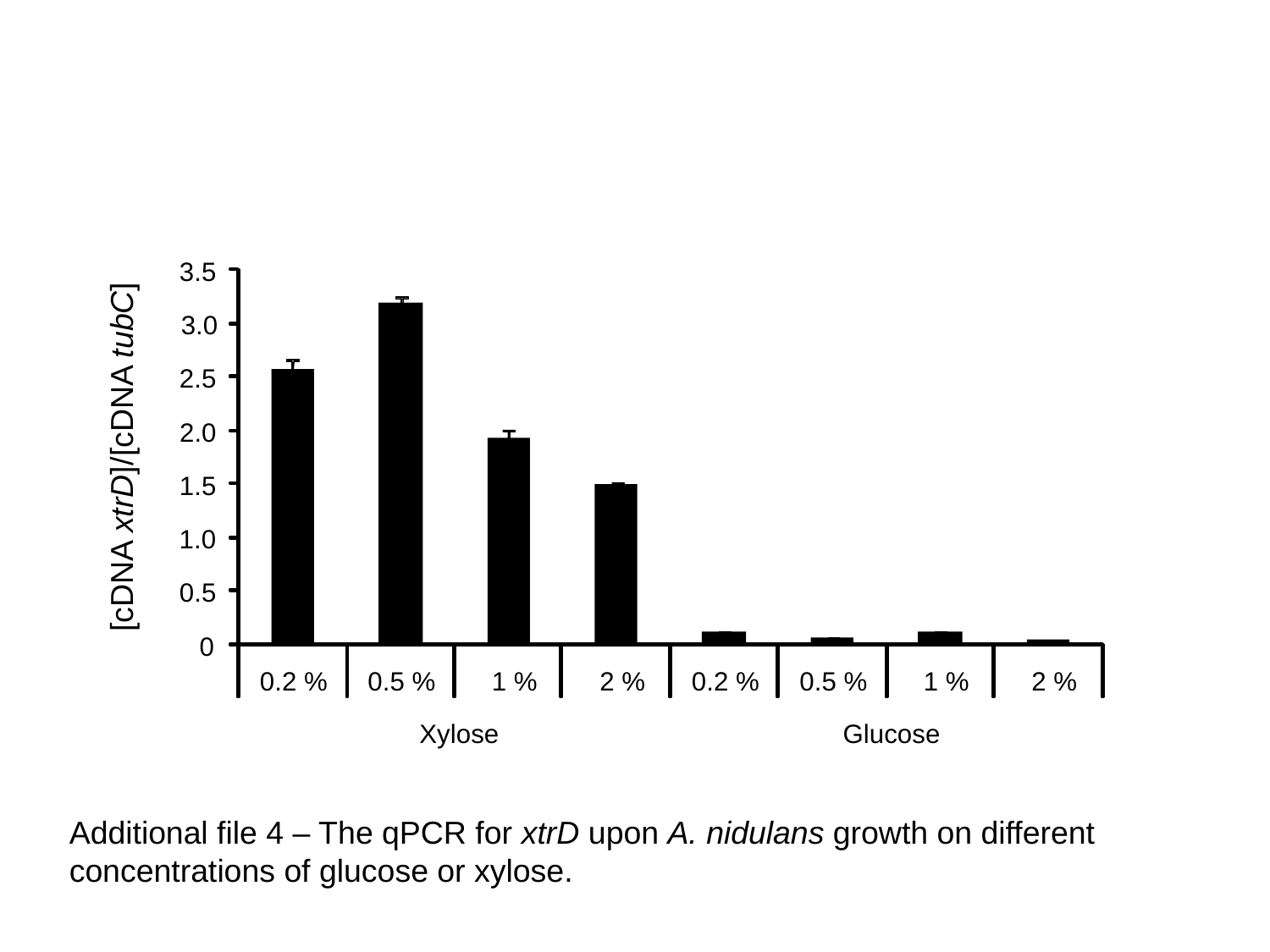

3.5
3.0
2.5
2.0
[cDNA xtrD]/[cDNA tubC]
1.5
1.0
0.5
0
0.2 %
0.5 %
1 %
2 %
0.2 %
0.5 %
1 %
2 %
Xylose
Glucose
Additional file 4 – The qPCR for xtrD upon A. nidulans growth on different
concentrations of glucose or xylose.
