# Supplementary material for: Functional characterization of a xylose transporter in Aspergillus nidulans
Source: Biotechnol Biofuels. 2014 Apr 1;7:46. doi: 10.1186/1754-6834-7-46 (PMC4021826; doi:10.1186/1754-6834-7-46)
Supplement: Additional file 5 — Primers used in this work. [file 1754-6834-7-46-S5.pdf]

Additional file 5 – Primers used in this work

tubCSybr FW	5’ AGCTGGCGGTAACAAATACG 3’
tubCSybr RV	5’ ACCTGATCCACCAATTCTGC 3’
AN3264Sybr FW	5’ TTTGAAAGGGACGGCAATAG 3’
AN3264SybrRV	5’ CGCCAACACCCTGAATAGAT 3’
AN0250Sybr FW	5’ CCTTGGCTCTTCAACTCGTC 3’
AN0250Sybr RV	5’ GTTTTTCGAAGACGCTCTGG 3’
AN6412Sybr FW	5’ TCGCCCTCTCTCTCACACTT 3’
AN6412Sybr RV	5’ AACTCTTCGCCTGGAACAGA 3’
AN2358Sybr FW	5’ TCCTCATCTTCGCCATTCTC 3’
AN2358Sybr RV	5’ CCCATACAGTGTCCCAAACC 3’
AN4148Sybr FW	5’ CAGCTTCATCCAGACGGAAT 3’
AN4148Sybr RV	5’ TCTCGAGCACAACAGGAATG 3’
AN0250 5F pRS426	5'GGTTTTCCCAGTCACGACGTAGCACCGCCTGAGGTCTCC 3'
AN02505RGFP RV	5' CTCCTTTACTCATTCCCCGTGTTCCTGCGTTCTTTGGCTCCACC 3'
AN0250 3F pyrG	5' GCATTGTTTGAGGCGAATTCTATTGGCTTCTGGTTTCATTG 3'
AN0250 3R pRS426	5' CAATGAAACCAGAAGCCAATAGAATTCGCCTCAAACAATGC 3'
AN0250 lev1	5' GAATTAATAAAAGTGTTCGCTCATGCGTTCTTTGGCTCCAC 3'
AN0250 lev2	5' GTTTTTTTAATTTTAATCAAAATGGGCGGCGCCGGCGATACC 3'
AN0250 spacer gfp FW lev	5' GTTCTTCTCCTTTACTCATTCCCCGTGTTCCTGCGTTCTTTGGCTCC 3'
