# Supplementary material for: Functional characterization of a xylose transporter in Aspergillus nidulans
Source: Biotechnol Biofuels. 2014 Apr 1;7:46. doi: 10.1186/1754-6834-7-46 (PMC4021826; doi:10.1186/1754-6834-7-46)
Supplement: Additional file 6 — Plasmids used in this work. [file 1754-6834-7-46-S6.pdf]

Additional file 6 – Plasmids used in this work

pRH195	pRS414 + P*HXT7*-*XKS1*-T*HXT7*	Reference [9]
pRH195m	pRS414 + P*HXT7*-*xrtD*-T*HXT7*	This work
pRH274	pRS416 + P*PGK*1-*XYL1*-T*PGK*1; P*ADH*1-*XYL2*-T*ADH*1; P*HXT7*-*XKS1*-T*HXT7*	Reference [9]
